# Investigation of the effects of P1 on HC-pro-mediated gene silencing suppression through genetics and omics approaches

**DOI:** 10.1186/s40529-020-00299-x

**Published:** 2020-08-03

**Authors:** Sin-Fen Hu, Wei-Lun Wei, Syuan-Fei Hong, Ru-Ying Fang, Hsin-Yi Wu, Pin-Chun Lin, Neda Sanobar, Hsin-Ping Wang, Margo Sulistio, Chun-Ta Wu, Hsiao-Feng Lo, Shih-Shun Lin

**Affiliations:** 1 Institute of Biotechnology, National Taiwan University, Taipei, 106 Taiwan; 2 Agricultural Biotechnology Research Center, Academia Sinica, Taipei, 115 Taiwan; 3 Center of Biotechnology, National Taiwan University, Taipei, 106 Taiwan; 4 Instrumentation Center, National Taiwan University, Taipei, 106 Taiwan; 5 Department of Horticulture and Landscape Architecture, National Taiwan University, Taipei, 106 Taiwan

**Keywords:** P1/HC-Pro, Viral suppressor, Posttranscriptional gene silencing, MicroRNA, Omics, Comparative network

## Abstract

**Background:**

Posttranscriptional gene silencing (PTGS) is one of the most important mechanisms for plants during viral infection. However, viruses have also developed viral suppressors to negatively control PTGS by inhibiting microRNA (miRNA) and short-interfering RNA (siRNA) regulation in plants. The first identified viral suppressor, P1/HC-Pro, is a fusion protein that was translated from potyviral RNA. Upon infecting plants, the P1 protein itself is released from HC-Pro by the self-cleaving activity of P1. P1 has an unknown function in enhancing HC-Pro-mediated PTGS suppression. We performed proteomics to identify P1-interacting proteins. We also performed transcriptomics that were generated from Col-0 and various P1/HC-Pro-related transgenic plants to identify novel genes. The results showed several novel genes were identified through the comparative network analysis that might be involved in P1/HC-Pro-mediated PTGS suppression.

**Results:**

First, we demonstrated that P1 enhances HC-Pro function and that the mechanism might work through P1 binding to VERNALIZATION INDEPENDENCE 3/SUPERKILLER 8 (VIP3/SKI8), a subunit of the exosome, to interfere with the 5**′**-fragment of the PTGS-cleaved RNA degradation product. Second, the AGO1 was specifically posttranslationally degraded in transgenic Arabidopsis expressing *P1/HC*-*Pro* of turnip mosaic virus (TuMV) (*P1/HC*^*Tu*^ plant). Third, the comparative network highlighted potentially critical genes in PTGS, including miRNA targets, calcium signaling, hormone (JA, ET, and ABA) signaling, and defense response.

**Conclusion:**

Through these genetic and omics approaches, we revealed an overall perspective to identify many critical genes involved in PTGS. These new findings significantly impact in our understanding of P1/HC-Pro-mediated PTGS suppression.

## Background

Posttranscriptional gene silencing (PTGS) includes the regulation of microRNA (miRNA) and short-interfering RNA (siRNA) in plant development. The DICER-LIKE 2 (DCL2)/DICER-LIKE 4 (DCL4)-mediated siRNA pathway is a major defense system that inhibits viral infection. However, different species of viruses have developed various suppressors to counteract the DCL2/4-mediated siRNA defense system, known as PTGS suppression, making these species capable of surviving and multiplying in the infected plants. Viral suppressors of PTGS not only suppress the siRNA defense system but also inhibit miRNA regulation, resulting in symptom development. Symptoms represent the misregulation of the miRNA phenomena, whereas a mutant virus has a defective suppressor that causes mild symptoms and has a limited inhibitory effect on miRNA-regulation (Kung et al. 2014; Wu et al. 2010).

Viral suppressors of PTGS have various approaches to interfere with miRNA biogenesis or miRNA regulation. For instance, 2b of the cucumber mosaic virus (CMV) Q strain and p19 of tomato busy stunt virus (TBSV) binds miRNA and siRNA to prevent those of small RNAs loading into AGO1 (Silhavy et al. 2002; Zhang et al. 2006). P0 of polerovirus has an F-box-like domain to trigger AGO1 degradation (Michaeli et al. 2019). P1/HC-Pro is the first identified viral suppressor of PTGS (Anandalakshmi et al. 1998; Kasschau and Carrington, 1998). HC-Pro is a highly conserved protein in potyvirus that plays a major role in PTGS suppression (Kasschau and Carrington, 1998; Kasschau et al. 2003; Kung et al. 2014; Valli et al. 2006). In contrast, P1 is a highly divergent protein that has variable sequences in each potyvirus. P1 of tobacco etch virus (TEV) can enhance the HC-Pro-mediated PTGS suppression; however, the mechanism is still unclear (Kasschau and Carrington, 1998; Martínez and Daròs, 2014; Valli et al. 2006). Martinez and Daròs (2014) demonstrated that P1 of TEV interacts with the 60S ribosomal subunit and enhances in vitro translation.

Previous studies demonstrated that *P1/HC*-*Pro* genes of zucchini yellow mosaic virus (ZYMV) and turnip mosaic virus (TuMV) suppressed miRNA regulation (Kung et al. 2014; Wu et al. 2010). Transgenic Arabidopsis expressing *P1/HC*-*Pro* of ZYMV (*P1/HC*^*Zy*^ plant) or *P1/HC*-*Pro* of TuMV (*P1/HC*^*Tu*^ plant) showed severe serrated and curling leaf phenotypes that are related to miRNA misregulation and viral symptom development (Kung et al. 2014; Wu et al. 2010). Moreover, the FRNK motif (highly conserved amino acid sequence) of HC-Pro in TuMV and ZYMV is necessary and sufficient for PTGS suppression (Kung et al. 2014; Wu et al. 2010). The miRNA misregulation in transgenic plant expressing viral suppressor gene, such as *2b*, *P1/HC*-*Pro*, and *P19*, is occurred by abnormal miRNA/miRNA* accumulation via an unknown mechanism, resulting in target RNA accumulation (Kasschau et al. 2003; Kung et al. 2014). Therefore, abnormal miRNA/miRNA* and target RNA accumulations are the molecular phenotypes of PTGS suppression.

In this study, we demonstrated that various potyviruses of P1 are necessary and sufficient to enhance HC-Pro PTGS suppression. Through high-throughput omics approaches, several critical genes that interact with P1 or are involved in PTGS were identified from immunoprecipitation (IP) and transcriptomic profiles. We also found that P1/HC-Pro of TuMV triggers Argonaute protein 1 (AGO1) posttranslational degradation. These critical genes offer new directions for further investigation of the PTGS and P1/HC-Pro-mediated suppression.

## Materials and methods

### Plant material and transgenic plants

*Arabidopsis thaliana* ecotype Col-0 and transgenic plants, *P1/HC*^*Tu*^ plant, and *P1/HC*^*Zy*^ plant (Wu et al. 2010) were used in this study. Arabidopsis seeds were surface sterilized and chilled at 4 °C for 2 days and then sown on Murashige and Skoog (MS) medium with/without suitable antibiotics. The seedlings were transferred into soil after 1 week of germination. All plants were grown at 24 °C in a growth room with 16 h of light/8 h of dark.

### Transgenic plant construction

For *P1/HC*^*Te*^ plant construction, the *P1/HC*-*Pro* gene of TEV was amplified from the pTEV-At17 plasmid (Agudelo-Romero et al. 2008) by polymerase chain reaction (PCR) with the primer set: PteP1 (5′-CACCATGGCACTCATCTT-3′) and MTEHC (5′-TCCAACATTGTAAGTTTT-3′). The PCR fragment was cloned into the pENTR/D-TOPO vector (Invitrogen) to generate pENTR-P1/HC^Te^. The pENTR vector was transferred into the pBCo-DC vector (Kung et al. 2014) using Gateway LR Clonase II Enzyme Mix (Thermo Fisher) to generate pBCo-P1/HC^Te^.

For *P1*^*Tu*^ plant construction, the TuMV infectious clone was used as a template to amplify the *P1*^*Tu*^ gene with the primer set: PtuP1/MTuP1 (5′-TCAAAAGTGCACAATCTT-3′), and the gene was then cloned into the pENTR and pBCo-DC vectors following the above procedures to generate pBCo-P1. For the *HC*^*Tu*^ plant resistant to Basta, the TuMV infectious clone was used as template to amplify the *HC*^*Tu*^ gene with the primer set: PTuHC (5′-CACCATGAGTGCAGCAGGAGCC-3′)/MTuHC, and it was then cloned into the pENTR and pBCo-DC vectors following the above procedures to generate the pBCo-HC^Tu^ fragment. An *Nhe*I site was introduced into the fusion form of the *P1HC*-*Pro* gene (*P1HC*^*Tu*−*FA*^) to generate a F_362_A substitution. Furthermore, the *P1* and *HC*-*Pro* genes were amplified from the TuMV infectious clone (Niu et al. 2006) and constructed under the 35S promoter to create the *P1*^*Tu*^ and *HC*^*Tu*^ plants, respectively. The pBCo-P1/HC^Te^, pBCo-P1^Tu^, pBCo-HC^Tu^, and pBCo-P1HC^Tu-FA^ binary vectors were transferred into Col-0 by the floral-dipping method with the *Agrobacterium tumefaciens* ABI strain to generate the *P1/HC*^*Te*^, *P1*^*Tu*^, and *HC*^*Tu*^ plants, respectively.

For recombined *P1/HC*-*Pro* transgenic plant construction, the infectious clones of TuMV, ZYMV, and TEV were used as templates to generate the recombinant *P1/HC*-*Pro* constructs. The P1 cleavage site in the recombined gene had to be preserved in the recombined constructs, and the constructs were cloned into the pBCo binary vector (Kung et al. 2014) for *Agrobacterium*-mediated flower-dipping transformation.

### Antibody production

For the TuMV P1 antibody, the N-terminus of the P1 (1-190 aa) of DNA fragment was amplified with the following primer sets PTuP1-NheI (5′-TATGGCTAGCATGGCAGTAGTTACATTCGC-3′)/MTuP1570-XhoI (5′-GGTGCTCGAGGCTCGCAGAGAGTCCTCCTC-3′). For the ZYMV P1 antibody, the N-terminus of the P1 (1-142 aa) DNA fragment was amplified with primer sets PZyP1- NheI (5′-TATGGCTAGCATGGCCTCAGTTATGATTGG-3′)/MZy426-XhoI (5′-GGTGCTCGAGCACTTCAGGTGGAAGAACAC-3′). For the TEV P1 antibody, the N-terminus of the P1 (1-133 aa) DNA fragment was amplified with primer sets PTeP1-NheI (5′-TATGGCTAGCATGGCACTCATCTTTGGCAC-3′)/MTe399-XhoI (5′-GGTGCTCGAGATCAACCTTTCTCTCGGTGT-3′).

For the TuMV HC-Pro antibody, the internal region of the HC-Pro (1-103 aa) of DNA fragment was amplified with primer sets PTuHC-NheI (5′-TATGGCTAGCACAGGGGAGGAATTCTCACA-3′)/MTuHC309-XhoI (5′-GGTGCTCGAGGATTGCAAGTTTCCGTGACC-3′). For the ZYMV HC-Pro antibody, the internal region of the HC-Pro (1-87 aa) DNA fragment was amplified with primer sets PZyHC-NheI (5′-TATGGCTAGCACACAGGCAACTCAGAATCT-3′)/MZyHC261-XhoI (5′-GGTGCTCGAGAGGATTTATCATAGCCTTGC-3′). For the TEV HC-Pro antibody, the internal region of the HC-Pro (1-99 aa) DNA fragment was amplified with primer sets PTeHC-NheI (5′-TATGGCTAGCACAGGGGCTGATCTCGAAGA-3′)/MTeHC297-XhoI (5′-GGTGCTCGAGTCTGCTACCCCTGATATGTT-3′).

All of the PCR fragments were digested with *Nhe*I and *Xho*I and then ligated with the same restriction enzyme-digested pET-28a vector to generate pET-P1^Tu^, pET-P1^Zy^, pET-P1^Te^, pET-HC^Tu^, pET-HC^Zy^, and pET-HC^Te^). All of the pET28 plasmids were transformed into the *E. coli* BL21 strain for recombinant protein expression. All recombinant proteins were purified by fast protein liquid chromatography (FPLC) (AKTApurifier, GE Healthcare). One milligram of recombinant protein with a 1 × volume of complete Freund’s adjuvant was injected into New Zealand white rabbits for the first injection. The following three injections consisted of 1 mg of protein mixed with a 1 × volume of incomplete Freund’s adjuvant. IgG purification was performed according to the protocol of Chiu et al. (2013). The IgG was collected after 4 injections for western blot detection.

### Immunoprecipitation and in-solution protein digestion

To identify the P1-interacting proteins, 250 mg of 10-day-old seedlings (*n *= 6) were homogenized with 1 mL IP buffer [25 mM Tris–HCl, pH 7.0, 150 mM NaCl, 1 mM EDTA, 5% glycerol, and a protease inhibitor (Roche)], followed by centrifugation for 10 min at 4 °C. IgG of α-P1^Tu^, α-P1^Zy^, and α-P1^Te^ was used for the in vivo IP. IP was performed by mixing 30 μl of washed Protein A Mag Sepharose^TM^ Xtra ferrite beads (GE), IgG (30 μl per IP reaction) and lysate. The IP reaction was carried out at 4 °C with gentle mixing for 3 h. The tube was then centrifuged at 300 g to pull-down the beads, which were washed three times with 0.3 mL wash buffer (25 mM Tris, 150 mM NaCl, 1 mM EDTA, 5% glycerol, 0.1% Triton-X-100, and a protease inhibitor) to remove nonspecific binding. Finally, the beads were resuspended in 50 μL elution buffer (0.1 M glycine, pH 2.0), and the reaction was mixed on a rotary at 4 °C for 10 min. A total of 10 μL of neutralization buffer (Tris–HCl, pH 8.0) was added to neutralize the reaction.

The proteins were dissolved in 6 M urea. A total of 15 μg of protein from each time point was used for in-solution digestion. Proteins were reduced by incubation with 10 mM dithiothreitol (DTT) for 1 h at 29 °C and alkylated by 55 mM iodoacetamide (IAA) at room temperature for 1 h. This step was quenched by 55 mM DTT at 29 °C for 45 min. The concentration of urea was diluted to 1 M before the sample was subjected to proteolysis. Protein digestion was performed overnight at 29 °C using mass spectrometry-grade modified trypsin (Promega) at a 1:50 trypsin/protein ratio. After overnight incubation, 0.1% TFA was added to stop the digestion. Finally, all remaining reagents from the in-solution digestion procedure were removed using a C18 stage tip.

### LC–MS/MS analysis

High-performance liquid chromatography with tandem mass spectrometry (LC–MS/MS) was performed on an Orbitrap Fusion Lumos Tribrid quadrupole-ion trap mass spectrometer (Thermo Fisher Scientific) in the Instrumentation Center of National Taiwan University. Peptides were separated on an Ultimate System 3000 NanoLC System (Thermo Fisher Scientific). Peptide mixtures were loaded onto a 75 μm inner diameter (ID), 25 cm length C18 Acclaim PepMap NanoLC column (Thermo Scientific) packed with 2 μm particles with a pore size of 100 Å. Mobile phase A was 0.1% formic acid in water, and mobile phase B was 100% acetonitrile with 0.1% formic acid. A segmented gradient was set over 90 min from 2% to 35% solvent B at a flow rate of 300 nl/min. Mass spectrometry analysis was performed in a data-dependent mode with full-MS (externally calibrated to a mass accuracy of < 5 ppm, and a resolution of 120,000 at *m/z *= 200), followed by high-energy collision activated dissociation (HCD)-MS/MS of the most intense ions in 3 s. HCD-MS/MS (resolution of 15,000) was used to fragment multiply charged ions within a 1.4 Da isolation window at a normalized collision energy of 32. An automatic gain control (AGC) target at 5e5 and 5e4 was set for MS and MS/MS analysis, respectively, with previously selected ions dynamically excluded for 180 s. The max injection time was 50 ms.

### Identification and quantitation of the proteome by label-free labeling methods

Quantitative proteomics was performed by label-free quantitative proteomic analysis. The raw MS/MS data were searched against the UniProt Knowledgebase/Swiss-Prot *Arabidopsis thaliana* protein database (Mar 2019 version) by using the Mascot 2.3 search algorithm via the Proteome Discoverer (PD) package (version 2.2, Thermo Scientific). The search parameters were set as follows: peptide mass tolerance, 10 ppm; MS/MS ion mass tolerance, 0.02 Da; enzyme set as trypsin and allowance of up to two missed cleavages; and variable modifications including oxidation on methionine, deamidation on asparagine and glutamine residues, and carbamidomethylation of cysteine residues. Peptides were filtered based on a 1% FDR. Protein quantification was computed by the abundance of ions extracted from the MS spectra of the corresponding peptides. The normalization method was set to the total peptide amount.

### Whole-transcriptome analysis

Total RNAs that were isolated from 10-day-old seedlings of Col-0, *P1*^*Tu*^, *HC*^*Tu*^, and *P1/HC*^*Tu*^ plants (*n *= 3) were used for whole-transcriptome deep sequencing by the High Throughput Sequencing Core of Academia Sinica. The sequencing was accomplished by paired-end (2 × 125) strand-specific HiSeq sequencing (Illumina). The transcriptome was analyzed by the ContigViews system (www.contigviews.bioagri.ntu.edu.tw) of the NGS core of National Taiwan University. For the ContigViews network analysis in this study, the twofold differentially expressed genes (DEGs) between Col-0 and *P1/HC*^*Tu*^ plants (*n* = 3) with an 80% passing rate were selected for the assay. Reads with twofold log_10_ FPKM values of genes under 1.14 were trimmed. At least 10 samples from Col-0, *P1*^*Tu*^, *HC*^*Tu*^, and *P1/HC*^*Tu*^ profiles (*n* = 3) were selected to calculate the Pearson correlation with a 0.95 threshold for positive relation and a 0.9 threshold for negative relation. Notable, parameter determination is according to the highlighted genes and network complexity. These parameters can generate the best network for data mining in ContigViews.

### Ethylene detection

Three-week-old Col-0 and *P1/HC*^*Tu*^ plants (*n *= 3) were individually sealed in the 1.5 L chambers at 24 °C with 16 h light/8 h dark. Ethylene gas samples in (1 mL) were withdrawn and collected at 4, 24, 48, and 72 h and were analyzed by GC-8A gas chromatography (Shimadzu) equipped with a flame ionization detector (FID).

## Results

### P1 enhances the severity of the HC-Pro-mediated serrated leaf phenotype and PTGS suppression

**Fig. 1:**
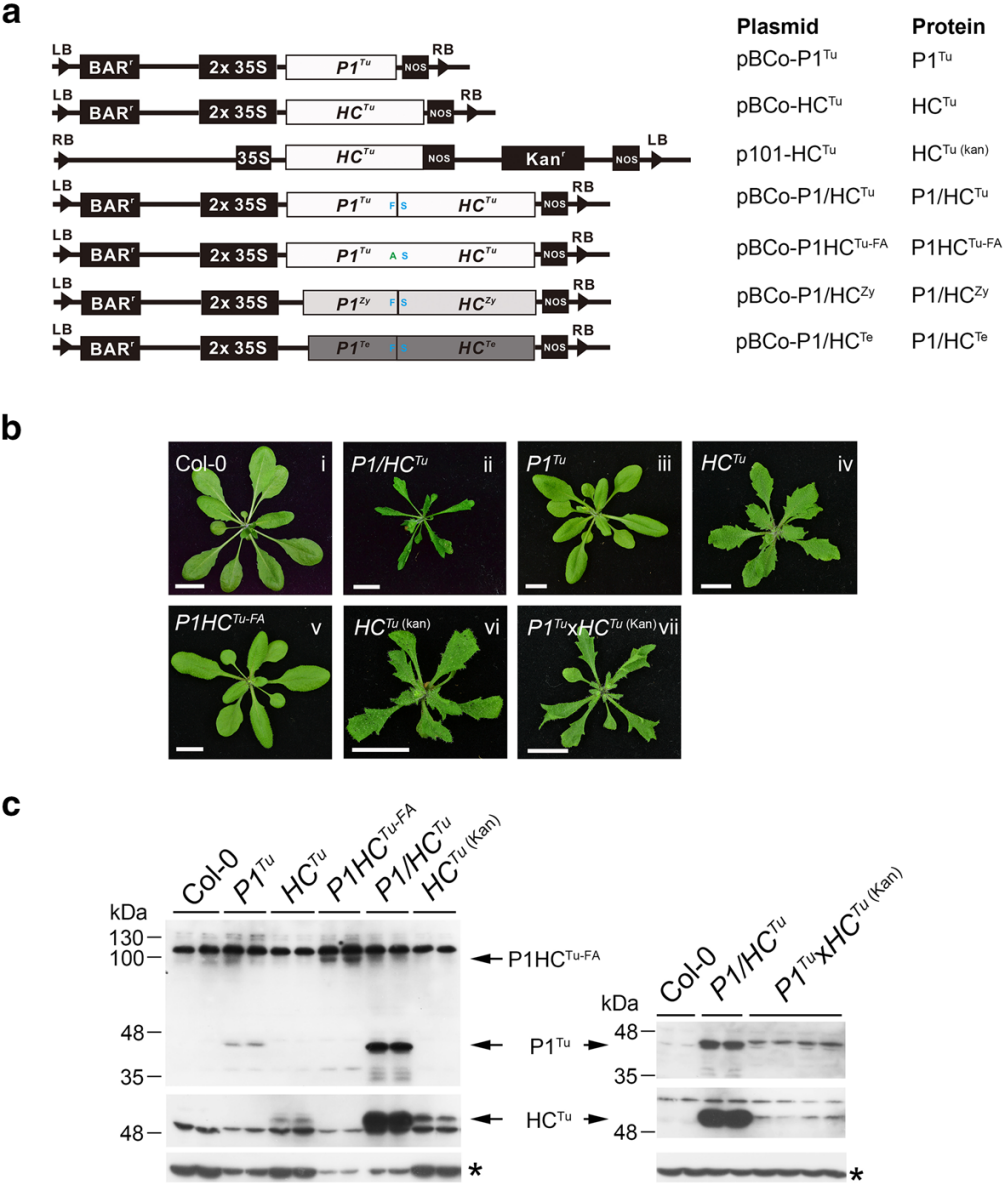
P1 enhances the HC^Tu^-mediated phenotype and HC^Tu^ suppression in miRNA-mediated regulation. **a** Schematic binary plasmids containing the various constructs that were used in this study. **b** Phenotypes of the different transgenic plants. The photographs were taken of 3-week-old seedlings. Bar, 1 cm. **c** Detection of P1 and HC-Pro of TuMV in various transgenic plants by western blotting. The asterisk (tubulin) is an internal control

To dissect the function of *P1*^*Tu*^ and *P1/HC*^*Tu*^ in PTGS suppression, we generated Arabidopsis transgenic lines expressing *P1*^*Tu*^ and *P1/HC*^*Tu*^ in combinations or individually (Fig. 1a, b). The *P1/HC*^*Tu*^ plants showed a severe serrated and curled leaf phenotype (Fig. 1b, panel ii). The translated P1/HC-Pro protein contains an F_362_/S_363_ cleavage site (Fig. 1a), which can generate separated P1 and HC-Pro proteins through P1 cleavage (Fig. 1c). The *P1*^*Tu*^ plant showed normal development similar to that of the Col-0 plants, whereas the *HC*^*Tu*^ plant showed mildly serrated leaves (Fig. 1b, panels iii and iv). In addition to the difference in the severity of the leaf phenotype, the size of the *HC*^*Tu*^ plant was larger than that of the *P1/HC*^*Tu*^ plant (Fig. 1b, panels ii and iv).

In addition, an F_362_A substitution at the F_362_/S_363_-P1 cleavage site produced a P1HC-Pro fusion protein (P1HC^Tu-FA^) (Fig. 1a, c). This transgenic *P1HC*^*Tu*-*FA*^ plant showed a normal phenotype (Fig. 1b, panel v). Furthermore, a kanamycin-resistant *HC*^*Tu*^ plant [*HC*^*Tu*^^(kan)^ plant] was generated for crossing with the *P1*^*Tu*^ plant (Basta resistant) (Fig. 1a). Similar to the *HC*^*Tu*^ plant, the *HC*^*Tu*^^(kan)^ plant showed mildly serrated leaves (Fig. 1b, panel vi). Interestingly, the *P1*^*Tu*^× *HC*^*Tu* (Kan)^ offspring showed severely serrated and curled leaves, but the *P1*^*Tu*^× *HC*^*Tu* (Kan)^ plant was larger than that of the *P1/HC*^*Tu*^ plant (Fig. 1b, panel vii). In addition, only the *P1/HC*^*Tu*^ plant showed high levels of the P1 and HC-Pro proteins, while the other lines, even the *P1*^*Tu*^× *HC*^*Tu* (Kan)^ plant, showed low levels of P1 and HC-Pro (Fig. 1c).

**Fig. 2:**
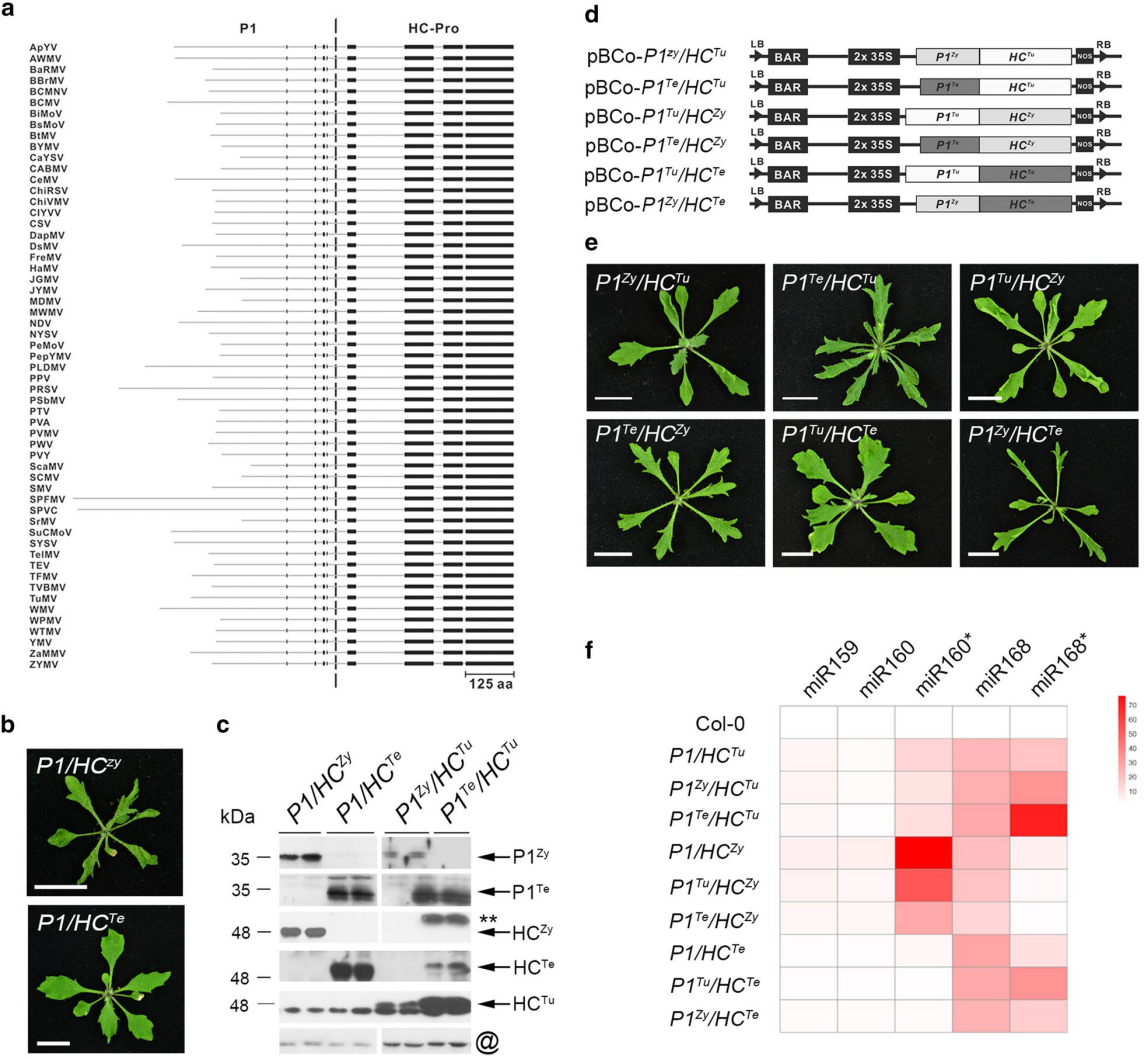
Variation in potyvirus P1/HC-Pro and recombined *P1/HC*-*Pro* plants. **a** Schematic diagram of P1 and HC-Pro amino acid sequence similarity among fifty-seven *Potyviruses*. The black boxes indicate conserved sequence regions. **b** The morphologic phenotype of *P1/HC*^*Zy*^ and *P1/HC*^*Te*^ plants. Bar, 1 cm. **c**. Detection of P1 and HC-Pro in *P1/HC*^*Zy*^, *P1/HC*^*Te*^, *P1*^*Zy*^*/HC*^*Tu*^, and *P1*^*Te*^*/HC*^*Tu*^ plants. The @ symbol indicates CDC2 as an internal control. Two-asterisk (**) indicate cross-reaction of the α-HC^Zy^ antibodies. **d** Schematic of the binary plasmids containing the various recombined *P1/HC*-*Pro* constructs used in this study. **e** Phenotypes of various recombinant *P1/HC*-*Pro* transgenic plants. The photographs were taken of 3-week-old seedlings. Bar, 1 cm. **f** Heatmap of abnormal miRNA and miRNA* accumulation in various recombinant *P1/HC*-*Pro* plants. The values of heatmap were convert from miRNA northern blot. Significant upregulation (Student’s *t* test; *P* value < 0.05) is labeled in red. Gray indicates differential expression that is not significant. The values on the right indicate the results of the log_2_ (each sample/Col-0) formula

We compared 57 potyvirus amino acid sequences of P1/HC-Pro (Fig. 2a). The alignment results showed that the sequence and length of the P1 protein in different potyviruses are highly diverse (Fig. 2a). Only the C-terminal protease activity site (black boxes) is conserved (Fig. 2a). In contrast, several conserved domains of HC-Pro were found in different species (Fig. 2a). To test whether the P1/HC-Pro from other potyviruses also induce serrated leaf phenotype, we generated Arabidopsis transgenic lines expressing P1/HC-Pro form ZYMV and TEV. *P1/HC*^*Zy*^ plants showed a severe serrated and curled leaf phenotype, whereas *P1/HC*^*Te*^ plants showed a minor serrated leaf phenotype (Fig. 2b). However, both plants had high levels of P1 and HC-Pro (Fig. 2c). The results indicated that the *P1/HC*-*Pro* genes of ZYMV and TEV can also trigger a serrated leaf phenotype.

The next question was whether the function of the HC-Pro from each virus requires the P1 from the same species. We generated 6 recombinant *P1/HC*-*Pro* plants in which HC-Pro was fused with a heterologous P1, namely, *P1*^*Zy*^*/HC*^*Tu*^, *P1*^*Te*^*/HC*^*Tu*^, *P1*^*Tu*^*/HC*^*Zy*^, *P1*^*Te*^*/HC*^*Zy*^, *P1*^*Tu*^*/HC*^*Te*^, and *P1*^*Zy*^*/HC*^*Te*^ (Fig. 2d). Except for *P1*^*Tu*^*/HC*^*Te*^ plants that show serrated leaves, the other 5 recombinant transgenic plants showed a severe serrated and curled leaf phenotype (Fig. 2e). The represented plants, *P1*^*Zy*^*/HC*^*Tu*^ and *P1*^*Te*^*/HC*^*Tu*^ plants, showed detectable P1 and HC-Pro expression (Fig. 2c). These results suggest that multiple *P1* genes have conserved functions in enhancing the HC-Pro-mediated serrated leaf phenotype.

### HC-Pro-mediated PTGS suppression

**Fig. 3:**
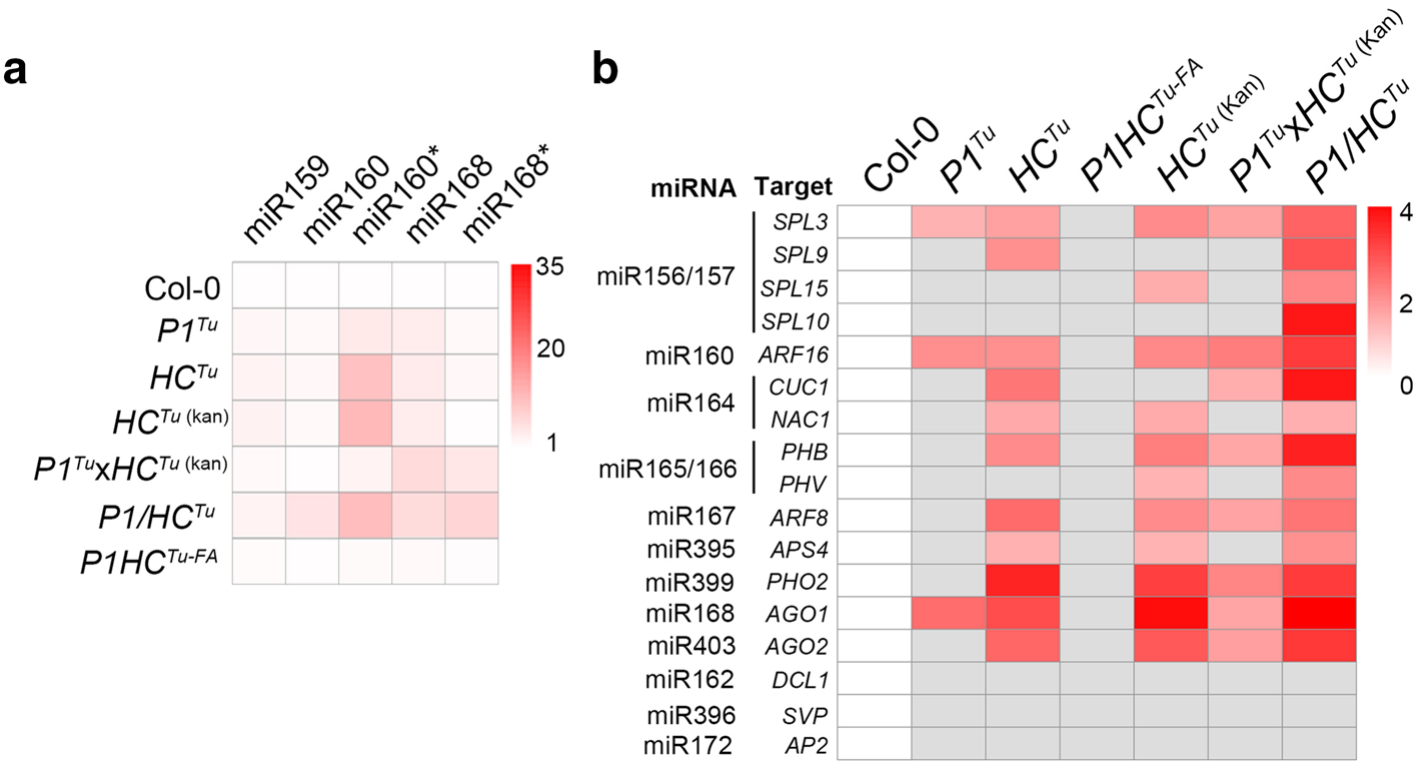
Abnormal accumulation of miRNA/miRNA*s and target mRNAs. **a** Heatmaps of miRNA and miRNA* and **b** miRNA target gene expression in various transgenic plants. The values of heatmap for miRNA were convert from miRNA northern blot. The values of heatmap for target RNAs were convert from transcriptome profiles. Significant upregulation (Student’s *t* test; *P* value < 0.05) is labeled in red. Gray indicates differential expression that is not significant. The values on the right indicate the results of the log2 (each sample/Col-0) formula

Previous studies demonstrated that an abnormal accumulation of miRNA and miRNA* occurs in several transgenic viral suppressor plants because suppressors interfere with miRNA biogenesis (Kasschau et al. 2003; Kung et al. 2014; Wu et al. 2010). Indeed, *P1/HC*^*Tu*^, *P1/HC*^*Zy*^, *P1/HC*^*Te*^, and 6 recombinant *P1/HC*-*Pro* plants showed abnormal miRNA/miRNA* accumulation (Fig. 2f). These data suggested that 3 species of viral P1/HC-Pro and recombinant P1/HC-Pro interfered with miRNA biogenesis. In addition, except for the *P1HC*^*Tu*-*FA*^ plant, all transgenic lines that contained HC^Tu^ showed abnormal miRNA and miRNA* accumulation (Fig. 3a), confirming that HC^Tu^ is the dominant player in PTGS suppression. Surprisingly, the *P1*^*Tu*^ plant also showed miRNA and miRNA* accumulation through an unknown mechanism (Fig. 3a). In addition to miRNA/miRNA* accumulation, miRNA targets were also upregulated in the transgenic plants because of miRNA misregulation (Kasschau et al. 2003; Kung et al. 2014; Wu et al. 2010). Transcriptome profiles also indicated that miRNA targets were upregulated in *HC*^*Tu*^, *HC*^*Tu* (kan)^, *P1*^*Tu*^× *HC*^*Tu*^, and *P1/HC*^*Tu*^ plants (Fig. 3b), suggesting that miRNA regulation was blocked by HC-Pro. However, *DICER*-*LIKE 1* (DCL1; miR162 target) and two translation inhibition genes, *APETALA 2* (*AP2*; miR172 target) and *SHORT VEGETATIVE PHASE* (*SVP*; miR396 target), showed no change in their transcript levels (Fig. 3b). Except for the *DCL1*, *AP2*, and *SVP* genes, the *P1/HC*^*Tu*^ plant suppressed most of the miRNA-target regulation (Fig. 3b). We conclude that the *P1/HC*^*Tu*^ plant has a stronger suppressive effect than the *HC*^*Tu*^ plants. In addition, the heterologous P1s have conserved function(s) in enhancing HC-Pro-mediated PTGS suppression.

### Host P1-interacting proteins are involved in PTGS

Because the recombinant P1/HC-Pro plants showed identical serrated leaf phenotypes and heterologous P1s could enhance HC-Pro-mediated PTGS suppression, we hypothesize that various P1 proteins have (a) conserved interacting protein(s) in Arabidopsis that enhance HC-Pro-mediated PTGS suppression. To identify the host P1-interacting proteins, the *P1/HC*^*Tu*^, *P1/HC*^*Zy*^, and *P1/HC*^*Te*^ plants were used for IP with α-P1^Tu^, α-P1^Zy^, and α-P1^Te^ antibodies, respectively. These IP eluates were analyzed by LC–MS/MS. We identified 101 cytoplasmic P1 of TuMV (P1^Tu^)-interacting proteins (Additional file 1: Data). Furthermore, we identified 56 cytoplasmic P1 of ZYMV (P1^Zy^)-interacting proteins and 20 cytoplasmic P1 of TEV (P1^Te^)-interacting proteins (Additional file 1: Data). Importantly, only one consensus cytoplasmic protein, VERNALIZATION INDEPENDENCE 3/SUPERKILLER8 (VIP3/SKI8; AT4G29830), was found in the IP profiles of 3 viral P1s (Table 1). VIP3/SKI8 is a subunit of the RNA exosome complex that is required for degradation of the RISC 5′-cleavage fragment (Branscheid et al. 2015; Orban and Izaurralde 2005). In contrast, 12 consensus cytoplasmic proteins were identified in the P1^Tu^ and P1^Zy^ IP profiles, whereas 10 consensus proteins were identified in the P1^Tu^ and P1^Te^ IP profiles (Table 1). Moreover, 5 consensus cytoplasmic proteins were found in the P1^Zy^ and P1^Te^ IP profiles (Table 1).

**Table 1:** The P1 interacting proteins

AGI	Protein name	Description	Interacting with		
			P1^Tu^	P1^Zy^	P1^Te^
AT4G29830	VIP3/SKI8	WD repeat-containing protein	+^a^	+	+
AT5G61780	TSN2	Ribonuclease TUDOR 2	+		
AT5G07350	TSN1	Ribonuclease TUDOR 1	+		
AT3G13300	VSC	Varicose	+		
AT2G15430	DdRp	DNA-directed RNA polymerases subunit 3	+		
AT3G18165	MOS4	Modifier of SNC1,4	+		
AT1G79280	NUA	Nuclear-pore anchor (NUA)	+		
AT5G53480	Importin	Importin subunit beta-1	+		
AT4G16143	Importin	Importin subunit alpha-2	+		
AT3G43300	BIG5	Brefeldin A-inhibited guanine nucleotide-exchange protein 5	+		
AT3G47810	VSP29	Vacuolar protein sorting-associated protein 29	+		
AT5G24780	VSP1	Vegetative storage protein 1	+	+	
AT1G52400		Beta-d-glucopyranosyl abscisate beta-glucosidase	+	+	
AT1G53310		Phosphoenolpyruvate carboxylase 1	+	+	
AT1G16460		Thiosulfate/3-mercaptopyruvate sulfurtransferase 2	+	+	
AT3G27300		Glucose-6-phosphate 1-dehydrogenase, cytoplasmic isoform 1	+	+	
AT2G23930		Probable small nuclear ribonucleoprotein G	+	+	
AT2G31390		Probable fructokinase-1	+	+	
AT3G62830		UDP-glucuronic acid decarboxylase 2	+	+	
AT5G03630		Monodehydroascorbate reductase 2	+	+	
AT3G52560		Ubiquitin-conjugating enzyme E2 variant 1D	+	+	
AT1G64520		26S proteasome non-ATPase regulatory subunit 8 homolog A	+	+	
AT1G08830	SOD1	Superoxide dismutse [Cu–Zn] 1	+		+
AT3G55620		Eukaryotic translation initiation factor 6-2	+		+
AT5G41220	GST	Glutathione S-transferase T3	+		+
AT4G24190		Endoplasmin homolog	+		+
AT5G42980		Thioredoxin H3	+		+
AT1G72730		Eukaryotic initiation factor 4A-3	+		+
AT1G77760		Nitrate reductase [NADH] 1	+		+
AT1G78370	GST	Glutathione S-transferase U20	+		+
AT3G61220		(+)-neomenthol dehydrogenase	+		+
AT3G06650		ATP-citrate synthase beta chain protein 1		+	+
AT5G49460		ATP-citrate synthase beta chain protein 2		+	+
AT5G44316		Putative UPF0051 protein ABCI9		+	+
AT3G44300		Nitrilase 2		+	+

^a^The protein was identified in the relevant P1 transgenic plants and marked as “+”

Next, we focused on P1^Tu^-interacting proteins because the *P1/HC*^*Tu*^ plant was the model used in this study. In the P1^Tu^ IP profile, two TUDOR-SN ribonucleases [(TSN1 (AT5G07350) and TSN2 (AT5G61780)] were uniquely identified 5 to 6 times in a total of 6 IP experiments with *P1/HC*^*Tu*^ plants (Table 1, and Additional file 2: Table S1). TSN1 and TSN2 have been suggested to be involved in the regulation of uncapping mRNA and localize to processing bodies (P-bodies) and stress granules (Yan et al. 2014). Therefore, whether P1^Tu^ could alter the function of TSN1 and TSN2 is an interesting project for the further investigation. Moreover, VARICOSE (VSC; AT3G13300) and MODIFIER OF SNC1,4 (MOS4; AT3G18165), which are involved in RNA regulation, were identified in the P1^Tu^ IP profile (Table 1 and Additional file 2: Table S1). We also identified the NUCLEAR-PORE ANCHOR (NUA; AT1G79280), two IMPORTIN subunits (AT5G53480 and AT4G16143), and BREFELDIN A-INHIBITED GUANINE NUCLEOTIDE-EXCHANGE PROTEIN 5 (BIG5; AT3G43300), which are involved in protein or nucleic acid transport between the nucleus and cytosol (Table 1 and Additional file 2: Table S1) (Xue et al. 2019). Moreover, VACUOLAR PROTEIN SORTING-ASSOCIATED PROTEIN 29 (VSP29; AT3G47810) was identified, which participates in vacuolar protein trafficking and vacuolar sorting receptor recycling (Table 1 and Additional file 2: Table S1) (Kang et al. 2012).

### P1 and HC-Pro cause differentially expressed host proteins in transgenic plants

**Fig. 4:**
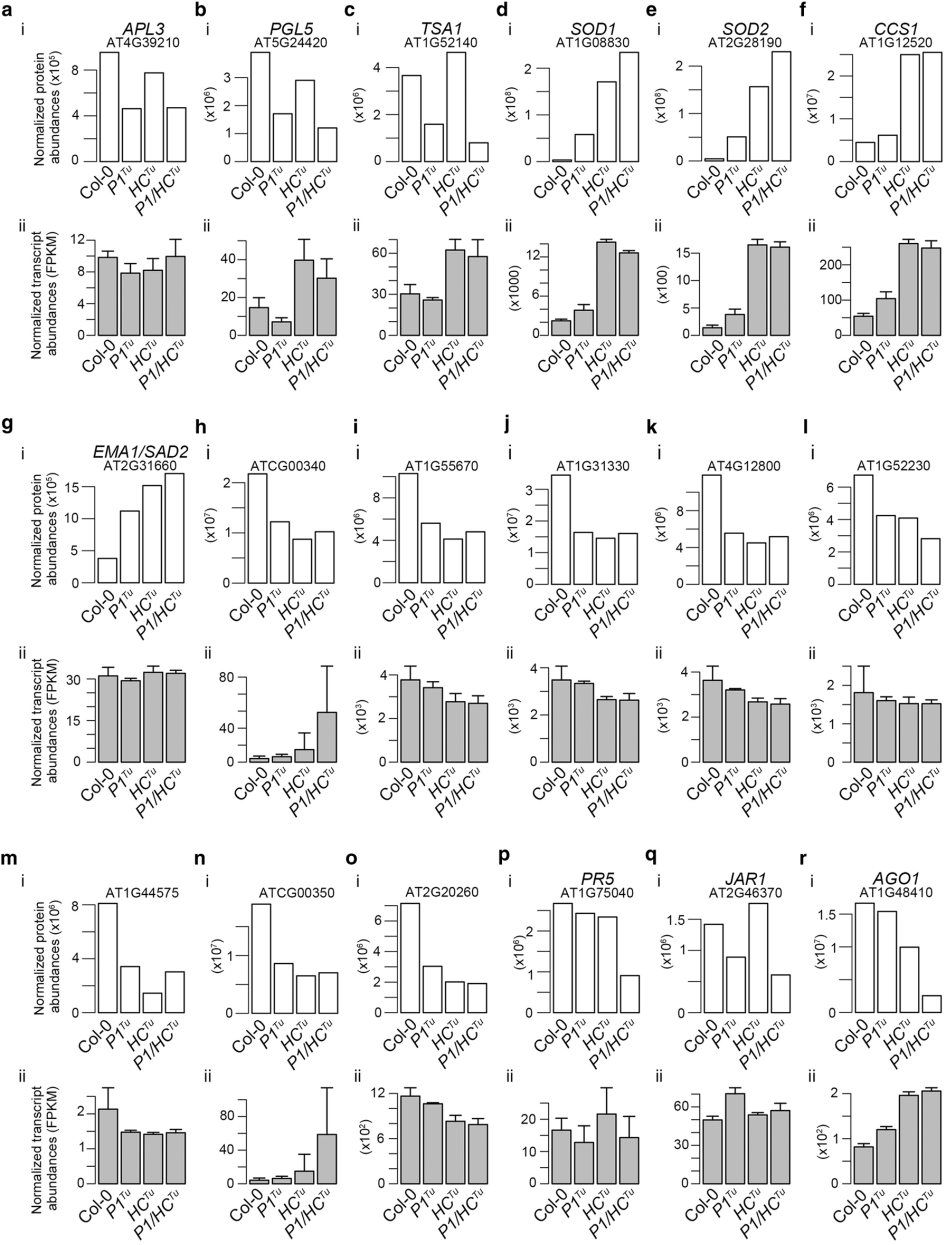
The protein and transcript levels of critical genes in various transgenic plants. **(a–r)** Genes that had a significant difference in protein levels (panel ii) between Col-0 and *P1/HC*^*Tu*^ plants were identified and their transcript levels were observed (panel i) in Col-0, *P1*^*Tu*^, *HC*^*Tu*^, and *P1/HC*^*Tu*^ plants. The fragments per kilobase of transcript per million (FPKM) were used to represent the normalized transcript expression. The bars represent standard deviations (*n *= 3). Normalized abundances were used to represent the protein amounts

We performed label-free proteomics to identify the differentially expressed host proteins between Col-0 and other transgenic plants. We identified 2757 Arabidopsis proteins in Col-0, *P1*^*Tu*^, *HC*^*Tu*^, and *P1/HC*^*Tu*^ plants (Additional file 1: Data). We found that ADP-GLUCOSE PYROPHOSPHORYLASE (APL3; AT4G39210), 6-PHOSPHOGLUCONOLACTONASE (PGL5; AT5G24420), and TONSOKU (TSK)-ASSOCIATING PROTEIN 1 (TSA1; AT1G52410) were decreased in *P1*^*Tu*^ and *P1/HC*^*Tu*^ plants compared to Col-0 plants but not decreased in *HC*^*Tu*^ plants (Fig. 4a–c, panel i). However, the transcript of *APL3* showed no significant difference among the various transgenic plants, whereas *PGL5* and *TSA1* were upregulated in *HC*^*Tu*^ and *P1/HC*^*Tu*^ plants compared with Col-0 plants (Fig. 4a–c, panel ii). APL3 is a starch biosynthesis enzyme, whereas PGL5 is a catalyzed enzyme in the oxidative pentose-phosphate pathway (OPPP) (Lansing et al. 2020; Liu et al. 2019). TSA1 is induced by methyl jasmonate (MeJA) and triggers endoplasmic reticulum (ER) body formation (Geem et al. 2019; Suzuki et al. 2005). These data indicated that the reduction in the protein levels of APL3, PGL5, and TSA1 occurred in a P1-dependent manner.

Next, we identified differentially expressed proteins between the *HC*^*Tu*^ and *P1/HC*^*Tu*^ plants. Nine proteins, including 2 superoxide dismutases [SOD1 (AT1G08830), and SOD2 (AT2G28190)], COPPER CHAPERONE FOR SOD1 (CCS1; AT1G12520), and ENHANCED MIRNA ACTIVITY 1/SUPER SENSITIVE TO ABA AND DROUGHT 2 (EMA1/SAD2; AT2G31660), were increased in *P1/HC*^*Tu*^ plants compared with *HC*^*Tu*^ plants (Fig. 4d–g, panel i). EMA1/SAD2 contains an importin-beta domain and negatively regulates in miRNA activity and is involved in abscisic acid (ABA) signaling (Cui et al. 2016; Panda et al. 2020; Wang et al. 2011).

In contrast, levels of 8 photosystem proteins (ATCG00340, AT1G55670, AT1G31330, AT4G12800, AT1G52230, AT1G44575, ATCG00350, and AT2G20260) were decreased in the *P1*^*Tu*^, *HC*^*Tu*^, and *P1/HC*^*Tu*^ plants compared with Col-0 (Fig. 4h–o, panel i). However, their transcript levels were not significantly different (Fig. 4h–o, panel ii). We also found that PATHOGENESIS-RELATED GENE 5 (PR5; AT1G75040) was decreased in *P1/HC*^*Tu*^ plants compared with Col-0 plants (Fig. 4P, panel i), whereas JASMONATE RESISTANT 1 (JAR1; AT2G46370) was decreased in *P1*^*Tu*^ and *P1/HC*^*Tu*^ plants (Fig. 4q, panel i). Similarly, the transcript levels of PR5 and JAR1 were not significantly different between plants (Fig. 4p, q, panel ii). In summary, many instances of posttranslational regulation occurred in the *P1*^*Tu*^, *HC*^*Tu*^, and *P1/HC*^*Tu*^ plants.

### The posttranscriptional and posttranslational regulation of miRNA targets in *P1/HC*^*Tu*^ plants

**Fig. 5:**
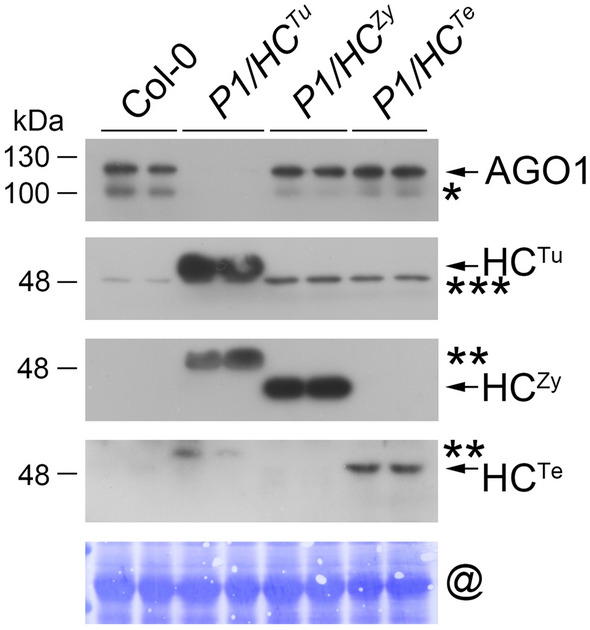
AGO1 protein detection in different viral *P1/HC*-*Pro* transgenic Arabidopsis plants. One asterisk (*) indicates the AGO1 isoform. Two-asterisk (**) indicates cross-reaction of the α-HC^Zy^ or α-HC^Te^ antibodies. Three-asterisk (***) indicates common bands. The @ symbol indicates RUBISCO as an internal control

CCS1 is involved in copper delivery, and SOD1 and SOD2 participate in Cu/Zn superoxide dismutase activities. The transcripts of these three genes are regulated by miR398 (Bouché 2010; Sunkar et al. 2006). However, there were high levels of *CCS1*, *SOD1*, and *SOD2* accumulation in the *HC*^*Tu*^ and *P1/HC*^*Tu*^ plants, which corresponded to their transcript levels, indicating P1/HC-Pro-mediated PTGS suppression (Fig. 4d–f, panel ii). Indeed, the transcript level of miR168-regulated *AGO1* (AT1G48410) was increased in *HC*^*Tu*^ and *P1/HC*^*Tu*^ plants compared with Col-0 (Fig. 4r, panel ii). Surprisingly, the level of AGO1 protein was decreased via an unknown mechanism in *HC*^*Tu*^ and *P1/HC*^*Tu*^ plants (Fig. 4r, panel i). The western blot data also indicated that the level of AGO1 was lower in *P1/HC*^*Tu*^ plants than in Col-0 plants but was similar to that in Col-0 plants, *P1/HC*^*Zy*^ and *P1/HC*^*Te*^ plants (Fig. 5). These data suggested that the P1/HC-Pro of TuMV has a specific ability to trigger the posttranslational degradation of AGO1.

### Comparative gene-to-gene network and transcriptome analysis

**Fig. 6:**
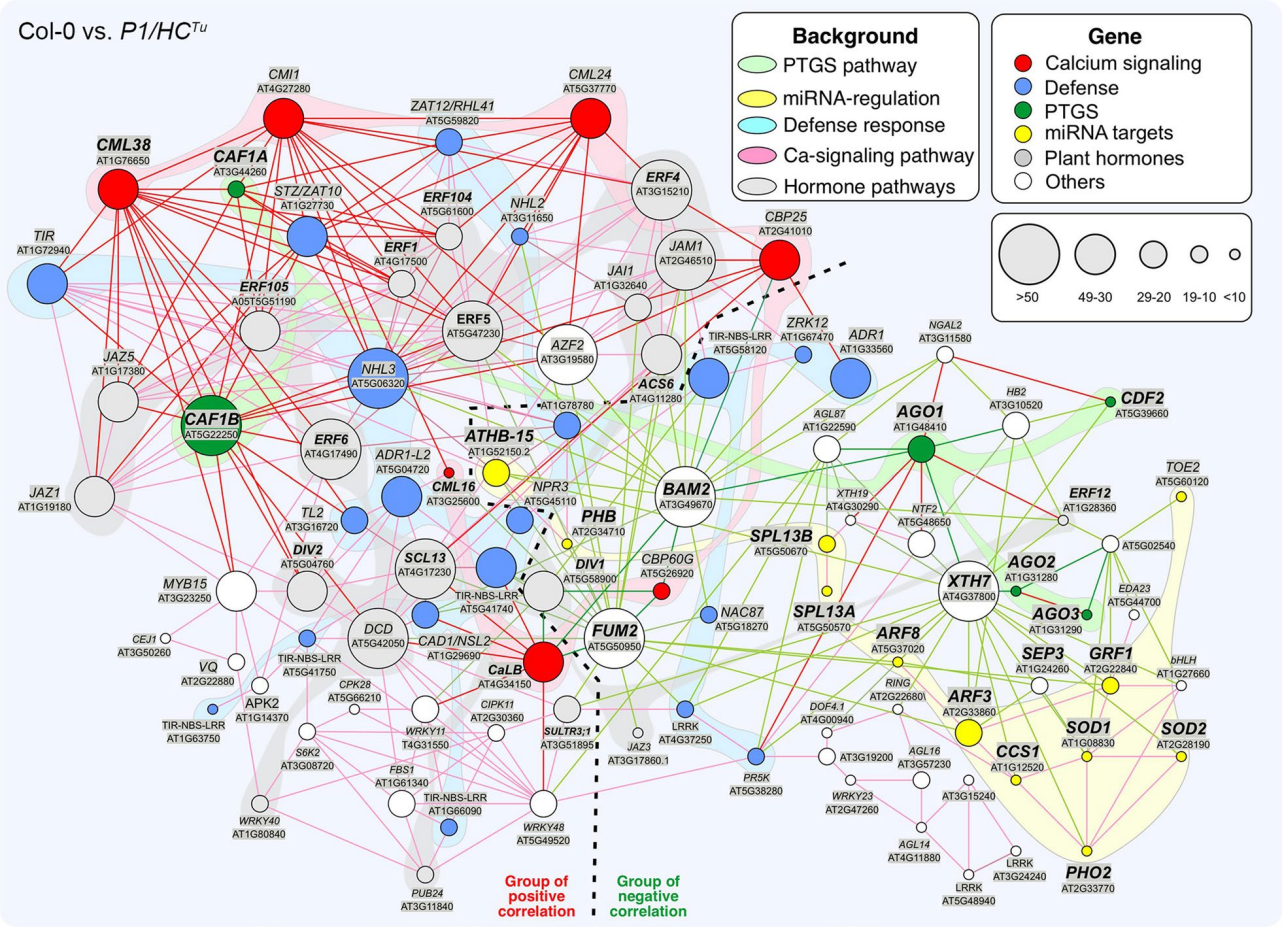
The gene-to-gene network of Col-0 vs. *P1/HC*^*Tu*^ plants. The gene profiles of twofold DEGs between Col-0 and *P1/HC*^*Tu*^ plants were used to generate the Pearson correlation network. The different circle sizes indicate the numbers of correlated genes. A positive correlation (> 0.95) between the two genes is indicated by a red line, whereas a green line indicates a negative correlation (< −0.9). The red circles indicate the genes involved in calcium signaling and are grouped with a red background. The blue circles indicate the genes involved in the defense response and are grouped with a blue background. The green circles indicate the genes involved in the PTGS pathway and are grouped with a green background. The yellow circles indicate the genes that are the miRNA targets and are grouped with a yellow background. The gray circles indicate the genes involved in the JA, ABA, and ethylene biosynthesis pathways and are grouped with a gray background

In the transcriptome analysis, we constructed a gene-to-gene correlation network to study PTGS suppression from a different perspective. First, we constructed a network for Col-0 vs. *P1/HC*^*Tu*^ plants in the ContigViews system. A list of twofold DGEs between Col-0 and *P1/HC*^*Tu*^ plants was used to generate a Pearson correlation network (Fig. 6). A group of positive correlations (red lines) and a group of negative correlations (green lines) were highlighted in the network (Fig. 6). The output of the network showed that *AGO1*, *AGO2* (AT1G31280), and *AGO3* (AT1G31290) were present in the group of negative correlations (Fig. 6). *AGO2* and *AGO3* were positively correlated with each other (red line) but had an indirect correlation with *AGO1* through *XYLOGLUCAN ENDOTRANSGLUCOSYLASE/HYDROLASE 7* (*XTH7*; AT4G37800) (Fig. 6). Notably, the transcripts of *AGO1*, *AGO2*, and *AGO3* were upregulated in the *HC*^*Tu*^ and *P1/HC*^*Tu*^ plants, but the *XTH7* transcripts were downregulated, suggesting that the AGOs and XTH7 might have opposite functions in PTGS (Fig. 4r, panel ii; Fig. 8a–c).

**Fig. 7:**
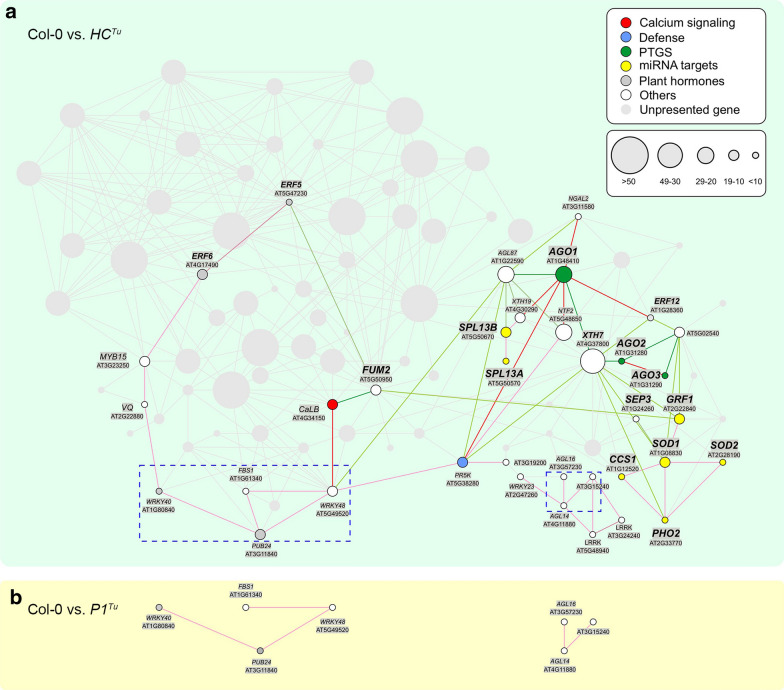
The comparative networks for Col-0 vs. *P1*^*Tu*^ and Col-0 vs. *HC*^*Tu*^ plants. **a** The gene profiles of twofold DEGs between Col-0 and *HC*^*Tu*^ plants were used to generate the Pearson correlation networks. **b** The gene profiles of twofold DEGs between Col-0 and P1^Tu^ plants were used to generate the network. The different circle sizes indicate the number of correlated genes. A positive correlation (> 0.95) between the two genes is indicated by a red line, whereas a green line indicates a negative correlation (< −0.9). The unpresented genes and correlation lines are indicated in gray

Next, we constructed two comparative networks, which were generated by a list of twofold DEGs between Col-0 and *HC*^*Tu*^ plants or between Col-0 and *P1*^*Tu*^ plants (Fig. 7a, b). The gene positions in the comparative networks were followed with the Col-0 vs. *P1/HC*^*Tu*^ network for comparison (Figs. 6 and 7). There were 97 genes in the Col-0 vs. *P1/HC*^*Tu*^ network (Fig. 6); however, there were only 36 genes showed up when we applied the same parameters in the Col-0 vs. *HC*^*Tu*^ network (Figs. 6 and  7a). In addition, the main genes involved in PTGS, such as *AGO1*, *AGO2*, *AGO3*, and *XTH7*, remained in the Col-0 vs. *HC*^*Tu*^ network (Fig. 7a). This suggested the presence of a basic network backbone in the HC^Tu^-mediated PTGS suppression that occurs without the effects of P1^Tu^. In contrast, the Col-0 vs. *P1*^*Tu*^ network only had 7 genes in 2 small groups that were also present in parts of the Col-0 vs. *HC*^*Tu*^ or Col-0 vs. *P1/HC*^*Tu*^ networks (Figs. 6 and  7). Moreover, *XTH7* had fewer than 49 connected genes in the Col-0 vs. *HC*^*Tu*^ network, whereas *XTH7* had 61 connections in the Col-0 vs. *P1/HC*^*Tu*^ network (Figs. 6 and  7a). These data indicated that the *XTH7* connection is variable in different networks and might play an important role in PTGS suppression. Overall, the comparative network analysis highlights the effects of P1^Tu^ on HC^Tu^-mediated PTGS suppression. This also explains why the *P1/HC*^*Tu*^ plant has a severe phenotype because of how many pathways were interfered with.

### Critical genes in the Col-0 vs. *P1/HC*^*Tu*^ network that are involved in PTGS

**Fig. 8:**
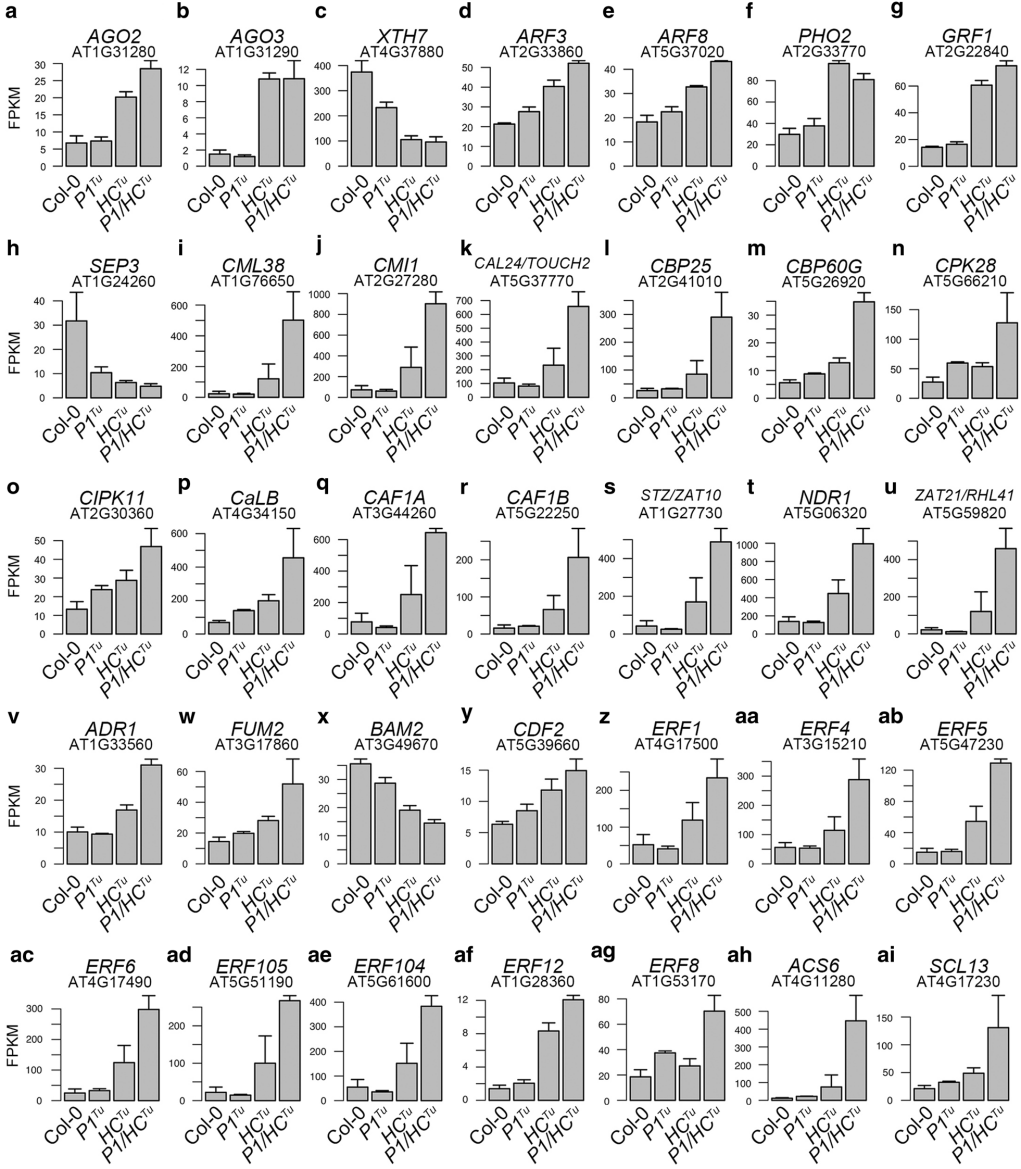
Transcript expression comparisons of critical genes in the networks. (**a-ai**) Genes that showed a significant connection, function, or position in the network were selected to demonstrate their transcript expression. The fragments per kilobase of transcript per million (FPKM) were used to represent the normalized transcript expression. The bars represent standard deviations (*n *= 3)

The importance of *XTH7* is not only in the number of gene connections it has or that it is connected with *AGO1* and *AGO2*; *XTH7* also had a negative correlation with several miRNA targets in the Col-0 vs. *P1/HC*^*Tu*^ network, such as 2 auxin response transcription factor genes [*ARF3* (AT2G33860) and *ARF8* (AT5G37020)], *PHOSPHATE 2* (*PHO2*; AT2G33770), *GROWTH*-*REGULATING FACTOR 1* (*GRF1*; AT2G22840), *CCS1*, *SOD1*, and *SOD2* (Fig. 5). However, *ARF3*, *ARF8*, *PHO2*, *GRF1*, *CCS1*, *SOD1*, and *SOD2* formed a positive correlation in the network (Fig. 6). These miRNA target transcripts were upregulated in *HC*^*Tu*^ and *P1/HC*^*Tu*^ plants because of PTGS suppression (Fig. 4e–f; panel ii; Fig. 8d–g). Moreover, *SEP3* (AT1G24260) showed negative correlations with *XTH7*, *ARF3*, *ARF8*, and *SOD1* (Fig. 7). In addition, *SEP3* transcript levels were lower in *P1*^*Tu*^, *HC*^*Tu*^, and *P1/HC*^*Tu*^ plants compared to Col-0 plants (Fig. 8h). Notably, SOD1 was shown to have a physical interaction with P1^Tu^ and P1^Te^ (Table 1) and was also highlighted in the network, suggesting the importance of SOD1 in PTGS suppression.

Four miRNA targets, including *TARGET OF EARLY ACTIVATION TAGGED 2* (*TOE2*; AT5G60120) and 2 squamosa promoter-binding protein-like genes [*SPL13A* (AT5G50570), and *SPL13B* (AT5G50670)], were also found in the group of negative correlation areas, whereas *ARABIDOPSIS THALIANA HOMEOBOX PROTEIN 15* (*ATHB*-*15*; AT1G52150) and *PHABULOSA* (*PHB*; AT2G34710) were found in the boundary between the positive and negative correlation groups (Fig. 6). Notably, miR172b regulated *TOE2* modules in regulating plant innate immunity (Zou et al. 2018). In the Col-0 vs. *P1/HC*^*Tu*^ network, *CYCLING DOF FACTOR 2* (*CDF2*; AT5G39660) and 2 carbon catabolite repressor 4 (CCR4)-associated factor genes [*CAF1A* (AT3G44260) and *CAF1B* (AT5G22250)] that are involved in RNA regulation were identified in the Col-0 vs. *P1/HC*^*Tu*^ network (Fig. 6). CAF1A and CAF1B catalyze mRNA deadenylation, whereas CDF2 interacts with DCL1 for miRNA biogenesis (Liang et al. 2009; Sun et al. 2015; Walley et al. 2010). These genes were also upregulated in *HC*^*Tu*^ and *P1/HC*^*Tu*^ plants (Fig. 8q, r, and y).

Eight calcium signaling genes were identified in the group of positive correlations in the network and were significantly upregulated in *P1/HC*^*Tu*^ plants (Figs. 6 and  8i–p). In the network, *CALMODULIN*-*LIKE* 24 (*CML24*; AT5G37770), and *CAM*-*BINDING PROTWIN 60*-*LIKE G* (*CBP60G*; AT5G26920) have significant functions in the regulation of autophagy and innate immunity, respectively (Qin et al. 2018; Tsai et al. 2013). In addition, jasmonic acid (JA) signaling and defense genes were highlighted in the positive correlation and their transcripts were upregulated in *HC*^*Tu*^ and *P1/HC*^*Tu*^ plants (Figs. 6 and  8q–v). In addition, the network indicated that *FUMARASE 2* (*FUM2*; AT5G50950) and *BARELY ANY MERISTEM 2* (*BAM2*; AT3G49670) were present in the boundary region between groups of positive and negative correlation (Fig. 6). BAM2 is a CLAVATA1-related receptor kinase and promotes the differentiation of stem cells on the meristem flank (DeYoung et al. 2006). *FUM2* transcripts were upregulated in *HC*^*Tu*^ and *P1/HC*^*Tu*^ plants, whereas *BAM2* transcripts were decreased (Fig. 8w and x). We also showed that *CDF2*, which is involved in miRNA biogenesis (Sun et al. 2015), is in the negative correlation group and is indirectly connected (negative correlation) to *AGO1* through *HB2* (AT3G10520) and *XTH7* (Fig. 6). The *CDF2* transcripts were upregulated in *HC*^*Tu*^ and *P1/HC*^*Tu*^ plants (Fig. 6y). The functions of critical genes in the Col-0 vs. *P1/HC*^*Tu*^ network are listed in Additional file 3: Table S2.

### The auxin, ethylene, and ABA signaling pathways in PTGS suppression

**Fig. 9:**
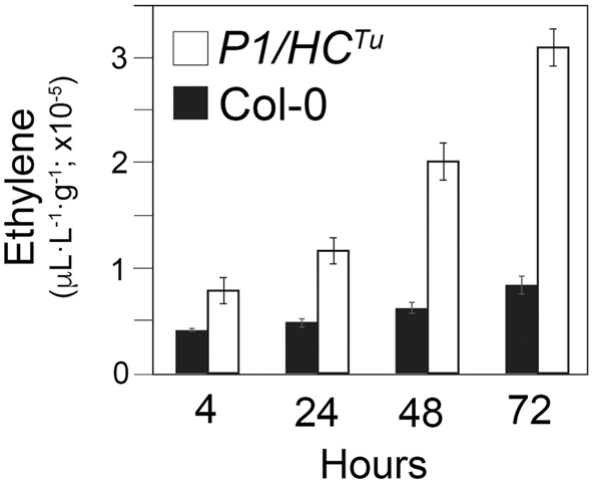
Time courses of endogenous ethylene detection in Col-0 and *P1/HC*^*Tu*^ plants. The bars represent standard deviations (*n *= 3)

The auxin response can induce ethylene and concomitantly trigger ABA biosynthesis (Hansen and Grossmann 2000). Importantly, the auxin, ethylene, and ABA signaling genes could be found in the Col-0 vs. *P1/HC*^*Tu*^ network (Fig. 6). MiRNA-regulated *ARF3* and *ARF8* targets are also auxin response genes, which were highly expressed in *HC*^*Tu*^ and *P1/HC*^*Tu*^ plants (Fig. 8d, e). In contrast, BAM2 expression is antagonistic with auxin transporters (Cecchetti et al. 2015) and its transcripts were downregulated in *HC*^*Tu*^ and *P1/HC*^*Tu*^ plants (Fig. 8x). Ethylene signaling genes, *1*-*AMINOCYCLOPROPANE*-*1*-*CARBOXYLIC ACID* (*ACC*) *SYNTHASE 6* (*ACS6*; AT4G11280), *SCARECROW*-*LIKE 13* (*SCL13*; AT4G17230), and 8 ethylene responsive element binding factors [*ERF1* (AT4G17500), *ERF4* (AT3G15210), *ERF5* (AT5G47230), *ERF6* (AT4G17490), *ERF105* (AT5G51190), *ERF104* (AT5G61600), *ERF12* (AT1G28360), *ERF8* (AT1G53170)] were present in the group of positive correlations and their transcripts were upregulated in *HC*^*Tu*^ and *P1/HC*^*Tu*^ plants (Figs. 6 and 8z-ai; and Additional file 3: Table S2). Moreover, the results from endogenous ethylene emission experiments showed higher ethylene levels were detected in *P1/HC*^*Tu*^ plants than those in Col-0 plants at each time point (Fig. 9). ABA signaling genes, *SULFATE TRANSPORTER 3;1* (*SULTR3;1*; AT3G51895), and 2 *DIVARICATA* genes [*DIV1* (AT5G58900), and *DIV2* (AT5G04760)] were also highlighted in the Col-0 vs. *P1/HC*^*Tu*^ network (Fig. 6) (Chen et al. 2019; Fang et al. 2018). These data suggested that P1/HC-Pro-mediated PTGS suppression also interferes with plant hormone signaling pathways.

## Discussion

### P1 enhances HC-Pro-mediated PTGS suppression

In *potyvirus*, P1 is a hypervariable protein with poorly understood its function. Previous studies suggested that P1 modulates virus replication, determines pathogenicity in a host-dependent manner, and triggers the host defense response (Maliogka et al. 2012; Pasin et al. 2014). In this study, we demonstrated that 3 viral P1s have a conserved function in enhancing HC-Pro-mediated PTGS suppression. From the perspective of P1-host protein interaction, VIP3/SKI8 turns over the 5′-fragment of RISC-cleaved target RNA, whereas TSN1, TSN2, and VSC are involved in mRNA decapping in stress granules and P-bodies (Branscheid et al. 2015; Deyholos et al. 2003; Gutierrez-Beltran et al. 2015; Sorenson et al. 2018; Xu and Chua 2009). Moreover, a MOS4 modifier, 2 IMPORTINs, and BIG5, which are involved in RNA splicing and RNA transportation, respectively, also interact with P1^Tu^ (Helizon et al. 2018; Kitakura et al. 2017; Luo et al. 2013; Xu et al. 2012). In addition, EMA1/SAD2 contains an importin-beta domain that negatively regulates miRNA activity (Wang et al. 2011). EMA1/SAD2 protein levels were upregulated in *P1*^*Tu*^, *HC*^*Tu*^, and *P1/HC*^*Tu*^ plants, but their transcript levels did not differ those in Col-0, suggesting P1 stabilized or increases EMA1/SAD2 levels to help inhibit miRNA regulation (Fig. 4g). Moreover, transcriptome data mining also showed that the *CAF1A/B* deadenylases and the *CDF2* zinc finger protein had a strong correlation with PTGS suppression. To summarize these findings, posttranscriptional RNA regulation occurs in stress granules and P-bodies, and many RNA regulatory components were identified among the proteins that interacted with P1 or were highlighted in the PTGS suppression network, which suggests that P1 is extremely vital for HC-Pro to enhance suppression.

Although P1 functions in regulating PTGS, it seems that P1 needs to be generated from the P1/HC-Pro fusion protein to have better enhance HC-Pro suppression. It is a cyclic effect in which lower levels of HC-Pro cause less efficiency in PTGS suppression, resulting in lower levels of HC-Pro. Indeed, the HC-Pro levels of *P1*^*Tu*^× *HC*^*Tu* (Kan)^ plants were similar to those of *HC*^*Tu*^ plants (Fig. 1c), suggesting ectopically expressed P1 did not have the same effect on enhancing HC-Pro as did P1 released from the fusion protein. Why must P1 be released from the fusion form to enhance HC-Pro ability? It is still unclear.

### P1/HC-Pro of TuMV specifically primes posttranslational AGO1 degradation

AGO1 degradation has been reported to be controlled by selective autophagy (Kobayashi et al. 2019; Li et al. 2019; Michaeli et al. 2019). The P0 viral suppressor of Polerovirus is thought to trigger autophagic AGO1 degradation (Michaeli et al. 2019). In our study, P1/HC-Pro of TuMV specifically triggered AGO1 posttranslational degradation, but the same effect was not observed in *P1/HC*^*Zy*^ and *P1/HC*^*Tu*^ plants, suggesting that P1/HC-Pro triggering AGO1 degradation does not occur in all potyviruses. In the other words, AGO1 degradation might not be essential for P1/HC-Pro-mediated PTGS suppression.

Autophagy works with vacuoles to allow for the degradation of large protein complexes. VSP29 is involved in the trafficking of vacuolar proteins and in the recycling of vacuolar sorting receptors and specifically interacts with P1^Tu^ (Table 1) (Kang et al. 2012). Moreover, CML24 interacts with AUTOPHAGY GENE 4b (ATG4b), which primes AUTOPHAGY GENE 8 (ATG8) by removing the C-terminus and exposing a glycine residue during autophagy (Tsai et al. 2013). CML24 was found to be present in the group of positive correlations of the PTGS network. Therefore, we implied that P1/HC-Pro of TuMV might also trigger AGO1 posttranslational degradation through autophagy.

### Network of HC-Pro-mediated PTGS suppression

The comparative gene correlation network provides a 4-dimensional perspective, which includes gene expression, gene correlation, position, and time course. This information is helpful to interpret and identify critical genes in pathways of interest. In the Co-0 vs. *HC*^*Tu*^ network, we identified a basic backbone network in HC^Tu^-mediated PTGS suppression. However, the effects of P1-enhanced HC-Pro suppression were highlighted in the Col-0 vs. *P1/HC*^*Tu*^ network upon comparing the two networks. The Col-0 vs. *P1/HC*^*Tu*^ network specifically highlighted the relationship between AGOs and viral resistance. Previous studies demonstrated that AGO2 and AGO3 upregulated to enhance the viral resistance (Alazem et al. 2017; Harvey et al. 2011; Zheng et al. 2019). *AGO2* is a target of miR403, which is negatively regulated by AGO1 (Harvey et al. 2011), suggesting the upregulation of AGO2 in response to AGO1 degradation in *P1/HC*^*Tu*^ plants. However, although we have no explanation for AGO3 upregulation, we assume that the AGO2/AGO3 antiviral system was activated and complemented AGO1 degradation. Indeed, AGO2 and AGO3 are directly positively correlated in the network.

Surprisingly, several miRNA targets, such as *CCS1*, *SOD1*, *SOD2*, *PHO2*, *ARF3*, and *GFR1*, showed a positive correlation in the network. These genes were indirectly negatively correlated with *AGO1* through *XTH7*. In addition, other miRNA targets, such as *TOE2*, *SPL13A/B*, *ATHB*-*15*, and *PHB* were also present in the network. *SPL13A* and *SPL13B* had direct positive correlations. ATHB-15 and PHB, which belong to the homeodomain-leucine zipper (HD-ZIP) transcription factor (TF) family, were also positively correlated. To summarize, the gene correlation network had significant accuracy in data mining.

Calcium signaling has been demonstrated to be involved in the suppression of gene silencing (Anandalakshmi et al. 2000; Nakahara et al. 2012). Anandalakshmi et al. (2000) demonstrated that the calmodulin-related protein (rgs-CaM) in tobacco interacts with HC-Pro and that it suppresses gene silencing similar to HC-Pro. Nakahara et al. (2012) demonstrated that tobacco rgs-CaM counterattacked various viral suppressors by binding to RNA-binding domains. In addition, rgs-CaM triggers autophagic viral suppressor degradation (Nakahara et al. 2012). Indeed, CML24 has physical interaction with ATG4b, suggesting that there is crosstalk between calcium signaling and autophagy (Tsai et al. 2013). CML24 was present in the group with a positive correlation, which was opposite to the AGOs that were present in the group with a negative correlation, suggesting that calcium signaling might counteract gene silencing.

We noted that several genes, such as *XTH7*, *FUM2*, and *BAM2*, had a significant number of connected genes (> 50 connected genes) (Fig. 6). XTH7 has been defined as a xyloglucan endotransglucosylase/hydrolase; however, little is known about its function in PTGS suppression. In addition, the cytosolic fumarase FUM2 is essential for Arabidopsis acclimation to low temperatures (Dyson et al. 2016). BAM2 is a CLAVATA1-related receptor kinase, and a little is known about its involvement in anther and meristem development (DeYoung et al. 2006; Hord et al. 2006). Although the functions of these genes were not explicitly linked with PTGS or defense, they were present in critical positions within the network with a large number of connected genes, which provides information for future research directions to investigate PTGS.

### Auxin and ethylene signaling in the serrated leaf phenotype

Current studies have indicated that the treatment with a high dose of auxin elicits endogenous ethylene production. In *P1/HC*^*Tu*^ plants, 3 auxin signaling genes (*ARF3*, *ARF8*, and *SUTR3;1*) were upregulated; therefore, we assume that ethylene was accumulated along with the increased expression of ethylene signaling genes. In addition, Hay et al. (2006) demonstrated that auxin can initiate marginal serrations in leaves, suggesting that the serrated leaf phenotype of *P1/HC*^*Tu*^ plants might be related to endogenous auxin accumulation.

## Conclusion

In this study, we used a transgenic plant approach to investigate the functions of P1 and HC-Pro. By mining high-throughput data from proteomic and transcriptomic profiles, P1-interacting proteins and critical genes in PTGS suppression were identified. Instead of traditional DEG identification, the comparative gene correlation network provides a four-dimensional perspective to identify critical genes, and provides new ideas and directions for further investigation. We believe plant molecular viology and plant molecular biology, like two hands, can be used together to efficiently investigate the PTGS mechanism.

## Supplementary information

**Additional file 1.** The profiles of protemoics.

**Additional file 2: Table S1.** The P1 interacting proteins.

**Additional file 3: Table S2.** Critical genes in Col-0 vs. *P1/HC*^*R*^ network.

## Data Availability

All data generated or analyzed in this study are in this published article.

## Abbreviations

ABA: Abscisic acid
ACC: 1-Aminocyclopropane-1-carboxylic acid
ACS6: 1-Aminocyclopropane-1-carboxylic acid synthase 6
AGC: Automatic gain control
AP2: Apetala 2
APL3: ADP-glucose pyrophosphorylase 3
ARF3: Auxin response transcription factor gene 3
ARF8: Auxin response transcription factor gene 8
ATG4b: Autophagy gene 4b
ATG8: Autophagy gene 8
ATHB-15: *Arabidopsis thaliana* homeobox protein 15
BAM2: Barely any meristem 2
BIG5: Brefeldin A-inhibited guanine nucleotide-exchange protein 5
CAF1A: Carbon catabolite repressor 4 (CCR4)-associated factor gene 1A
CAF1B: Carbon catabolite repressor 4 (CCR4)-associated factor gene 1B
CBP60G: Cam-binding protwin 60-like G
CCS1: Copper chaperone for SOD1
CDF2: Cycling dof factor 2
CML24: Calmodulin-like 24
Col-0: Columbia
DCL1: Dicer-like 1
DCL2: Dicer-like 2
DCL4: Dicer-like 4
DEG: Differentially expressed genes
DIV1: DIVARICATA gene 1
DIV2: DIVARICATA gene 2
DTT: Dithiothreitol
EMA1/SAD2: Enhanced MIRNA activity 1/super sensitive to ABA and drought 2
ER: Endoplasmic reticulum
ERF: Ethylene responsive element binding factors
FID: Flame ionization detector
FPKM: Fragments per kilobase of transcript per million
FPLC: Fast protein liquid chromatography
FUM2: FUMARASE 2
GRF1: Growth-regulating factor 1
HD-ZIP: Homeodomain-leucine zipper
IAA: Iodoacetamide
ID: Inner diameter
IP: Immunoprecipitation
JA: Jasmonic acid
JAR1: Jasmonate resistant 1
MeJA: Methyl jasmonate
miRNA: microRNA
MOS4: Modifier of SNC1: 4
MS: Murashige and Skoog
NUA: Nuclear-pore anchor
OPPP: Oxidative pentose-phosphate pathway
P-bodies: Processing bodies
PCR: Polymerase chain reaction
PD: Proteome discoverer
PGL5: 6-phosphogluconolactonase 5
PHB: Phabulosa
PHO2: Phosphate 2
PR5: Pathogenesis-related gene 5
PTGS: Posttranscriptional gene silencing
rgs-CaM: Calmodulin-related protein
RISC: RNA-induced silencing complex
SCL13: Scarecrow-like 13
siRNA: Short-interfering RNA
SOD1: Superoxide dismutase 1
SOD2: Superoxide dismutase 2
SPL13A: Squamosa promoter-binding protein-like gene 13A
SPL13B: Squamosa promoter-binding protein-like gene 13B
SULTR3;1: Sulfate transporter 3;1
SVP: Short vegetative phase
TEV: Tobacco etch virus
TF: Transcription factor
TOE2: Target of early activation tagged 2
TSA1: Tonsoku (TSK)-associating protein 1
TSN1: TUDOR-SN ribonucleases 1
TSN2: TUDOR-SN ribonucleases 2
TuMV: Turnip mosaic virus
VIP3/SKI8: Vernalization Independence 3/superkiller 8
VSC: Varicose
VSP29: Vacuolar protein sorting-associated protein 29
XTH7: Xyloglucan endotransglucosylase/hydrolase 7
ZYMV: Zucchini yellow mosaic virus
