# Supplementary material for: Investigation of the effects of P1 on HC-pro-mediated gene silencing suppression through genetics and omics approaches
Source: Bot Stud. 2020 Aug 3;61:22. doi: 10.1186/s40529-020-00299-x (PMC7399735; doi:10.1186/s40529-020-00299-x)
Supplement: Supplementary file 2 — Additional file 2: Table S1. The P1 interacting proteins. [file 40529_2020_299_MOESM2_ESM.docx]

**Additional file 2: Table S1. The P1 interacting proteins.**

| Antibodies | AGI | Gene Name | Detecting times |
| --- | --- | --- | --- |
| α-TuP1 |  | TuP1 | 6/6 |
|  | AT5G61780 | Ribonuclease TUDOR 2 (TSN2) | 6/6 |
|  | AT5G07350 | Ribonuclease TUDOR 1 (TSN1) | 5/6 |
|  | AT3G09440 | Heat shock 70 kDa protein 3 | 4/6 |
|  | AT5G24780 | Vegetative storage protein 1 | 4/6 |
|  | AT1G52400 | Beta-D-glucopyranosyl abscisate beta-glucosidase | 3/6 |
|  | AT1G63000 | Bifunctional dTDP-4-dehydrorhamnose 3,5-epimerase/dTDP-4-dehydrorhamnose reductase | 3/6 |
|  | AT2G30110 | Ubiquitin-activating enzyme E1 1 | 3/6 |
|  | AT4G01480 | Soluble inorganic pyrophosphatase 5 | 3/6 |
|  | AT1G53310 | Phosphoenolpyruvate carboxylase 1 | 2/6 |
|  | AT3G16470 | Jacalin-related lectin 35 | 2/6 |
|  | AT2G29360 | Tropinone reductase homolog At2g29360 | 2/6 |
|  | AT4G24190 | Endoplasmin homolog | 2/6 |
|  | AT5G64370 | Beta-ureidopropionase | 2/6 |
|  | AT1G16460 | Thiosulfate/3-mercaptopyruvate sulfurtransferase 2 | 2/6 |
|  | AT5G50370 | Adenylate kinase 3 | 2/6 |
|  | AT2G18230 | Soluble inorganic pyrophosphatase 2 | 2/6 |
|  | AT5G42980 | Thioredoxin H3 | 2/6 |
|  | AT3G27300 | Glucose-6-phosphate 1-dehydrogenase, cytoplasmic isoform 1 | 2/6 |
|  | AT3G46010 | Actin-depolymerizing factor 1 | 2/6 |
|  | AT5G26000 | Myrosinase 1 | 2/6 |
|  | AT2G23930 | Probable small nuclear ribonucleoprotein G | 2/6 |
|  | AT5G54960 | Pyruvate decarboxylase 2 | 2/6 |
|  | AT1G72730 | Eukaryotic initiation factor 4A-3 | 1/6 |
|  | AT5G64760 | 26S proteasome non-ATPase regulatory subunit 12 homolog B | 1/6 |
|  | AT5G64761 | Ran-binding protein M homolog | 1/6 |
|  | AT5G64762 | Protein arginine N-methyltransferase 1.1 | 1/6 |
|  | AT5G64763 | Probable histone-arginine methyltransferase 1.4 | 1/6 |
|  | AT5G64764 | Probable pyruvate kinase, cytosolic isozyme | 1/6 |
|  | AT5G64765 | Senescence-associated protein 13 | 1/6 |
|  | AT5G64766 | Tropinone reductase homolog At2g29290 | 1/6 |
|  | AT5G64767 | Tropinone reductase homolog At2g29370 | 1/6 |
|  | AT5G64768 | Tropinone reductase homolog At5g06060 | 1/6 |
|  | AT5G64769 | Coatomer subunit beta'-1 | 1/6 |
|  | AT5G64770 | Ribose-phosphate pyrophosphokinase 4 | 1/6 |
|  | AT3G47810 | Vacuolar protein sorting-associated protein 29 (VSP29) | 1/6 |
|  | AT5G64773 | 26S proteasome non-ATPase regulatory subunit 7 homolog A | 1/6 |
|  | AT5G47890 | NADH dehydrogenase [ubiquinone] 1 alpha subcomplex subunit 2 | 1/6 |
|  | ATCG00180 | DNA-directed RNA polymerase subunit beta | 1/6 |
|  | AT1G70830 | MLP-like protein 28 | 1/6 |
|  | AT5G05010 | Clathrin adaptor complexes medium subunit | 1/6 |
|  | AT1G77760 | Nitrate reductase [NADH] 1 | 1/6 |
|  | AT1G79280 | Nuclear-pore anchor (NUA) | 1/6 |
|  | AT5G27640 | Eukaryotic translation initiation factor 3 subunit B | 1/6 |
|  | AT1G13320 | Serine/threonine-protein phosphatase 2A 65 kDa regulatory subunit A gamma isoform | 1/6 |
|  | AT5G53480 | Importin subunit beta-1 | 1/6 |
|  | AT4G16143 | Importin subunit alpha-2 | 1/6 |
|  | AT1G01050 | Soluble inorganic pyrophosphatase 1 | 1/6 |
|  | AT5G63400 | Adenylate kinase 4 | 1/6 |
|  | AT1G70890 | MLP-like protein 43 | 1/6 |
|  | AT2G31390 | Probable fructokinase-1 | 1/6 |
|  | AT5G12110 | Elongation factor 1-beta 1 | 1/6 |
|  | AT1G61570 | Mitochondrial import inner membrane translocase subunit TIM13 | 1/6 |
|  | AT1G65980 | Peroxiredoxin-2B | 1/6 |
|  | AT5G43060 | Probable cysteine protease RD21B | 1/6 |
|  | AT1G02920 | Glutathione S-transferase F7 | 1/6 |
|  | AT3G62830 | UDP-glucuronic acid decarboxylase 2 | 1/6 |
|  | AT1G78370 | Glutathione S-transferase U20 | 1/6 |
|  | AT2G40840 | 4-alpha-glucanotransferase DPE2 | 1/6 |
|  | AT2G44490 | Beta-glucosidase 26, peroxisomal | 1/6 |
|  | AT1G79500 | 2-dehydro-3-deoxyphosphooctonate aldolase 1 | 1/6 |
|  | AT3G61220 | (+)-neomenthol dehydrogenase | 1/6 |
|  | AT1G75950 | SKP1-like protein 1A | 1/6 |
|  | AT5G03630 | Monodehydroascorbate reductase 2 | 1/6 |
|  | AT2G29530 | Mitochondrial import inner membrane translocase subunit TIM10 | 1/6 |
|  | AT3G18060 | Actin-interacting protein 1-2 | 1/6 |
|  | AT3G29250 | Short-chain dehydrogenase reductase 4 | 1/6 |
|  | AT3G13300 | **VARICOSE (VSC)** | 1/6 |
|  | AT3G02260 | Auxin transport protein BIG | 1/6 |
|  | AT5G42970 | COP9 signalosome complex subunit 4 | 1/6 |
|  | AT3G55620 | Eukaryotic translation initiation factor 6-2 | 1/6 |
|  | AT3G18165 | Modifier of SNC1,4 (MOS4) | 1/6 |
|  | AT2G39990 | Eukaryotic translation initiation factor 3 subunit F | 1/6 |
|  | AT5G41220 | Glutathione S-transferase T3 | 1/6 |
|  | AT4G29830 | WD repeat-containing protein VIP3 | 1/6 |
|  | AT2G15430 | DNA-directed RNA polymerases II, IV and V subunit 3 | 1/6 |
|  | AT3G43300 | Brefeldin A-inhibited guanine nucleotide-exchange protein 5 | 1/6 |
|  | AT1G11475 | DNA-directed RNA polymerases II, IV and V subunit 10 | 1/6 |
|  | AT3G52560 | Ubiquitin-conjugating enzyme E2 variant 1D | 1/6 |
|  | AT2G38750 | Annexin D4 | 1/6 |
|  | AT1G64520 | 26S proteasome non-ATPase regulatory subunit 8 homolog A | 1/6 |
|  | AT4G37880 | Protein RMD5 homolog | 1/6 |
|  | AT1G01300 | Aspartyl protease family protein 2 | 1/6 |
|  | AT1G35580 | Alkaline/neutral invertase CINV1 | 1/6 |
|  | AT3G55590 | Probable mannose-1-phosphate guanylyltransferase 2 | 1/6 |
|  | AT1G08830 | Superoxide dismutase [Cu-Zn] 1 | 1/6 |
|  | AT1G52070 | Jacalin-related lectin 10 | 1/6 |
|  | AT5G14800 | Pyrroline-5-carboxylate reductase | 1/6 |
|  | AT2G28720 | Histone H2B.3 | 1/6 |
| α-ZyP1 |  | ZYP1 | 6/6 |
|  | AT5G25230 | 109 kDa U5 small nuclear ribonucleoprotein component GFL | 4/6 |
|  | AT2G38540 | Non-specific lipid-transfer protein 1 | 3/6 |
|  | AT5G25980 | Myrosinase 2 | 3/6 |
|  | AT3G06650 | ATP-citrate synthase beta chain protein 1 | 3/6 |
|  | AT5G24780 | Vegetative storage protein 1 | 3/6 |
|  | AT2G35390 | Ribose-phosphate pyrophosphokinase 1, chloroplastic | 2/6 |
|  | AT5G52640 | Heat shock protein 90-1 | 2/6 |
|  | AT1G62020 | Coatomer subunit alpha-1 | 2/6 |
|  | AT1G63180 | Bifunctional UDP-glucose 4-epimerase and UDP-xylose 4-epimerase 3 | 2/6 |
|  | AT1G76550 | Pyrophosphate--fructose 6-phosphate 1-phosphotransferase subunit alpha 2 | 2/6 |
|  | AT3G14940 | Phosphoenolpyruvate carboxylase 3 | 2/6 |
|  | AT1G17720 | Serine/threonine protein phosphatase 2A 55 kDa regulatory subunit B beta isoform | 2/6 |
|  | AT5G49460 | ATP-citrate synthase beta chain protein 2 | 2/6 |
|  | AT3G44300 | Nitrilase 2 | 1/6 |
|  | AT3G16460 | Jacalin-related lectin 34 | 1/6 |
|  | AT1G52400 | Beta-D-glucopyranosyl abscisate beta-glucosidase | 1/6 |
|  | AT1G64520 | 26S proteasome non-ATPase regulatory subunit 8 homolog A | 1/6 |
|  | AT1G16460 | Thiosulfate/3-mercaptopyruvate sulfurtransferase 2 | 1/6 |
|  | AT5G22300 | Bifunctional nitrilase/nitrile hydratase NIT4 | 1/6 |
|  | AT1G72680 | Probable cinnamyl alcohol dehydrogenase 1 | 1/6 |
|  | AT2G23930 | Probable small nuclear ribonucleoprotein G | 1/6 |
|  | AT1G65960 | Glutamate decarboxylase 2 | 1/6 |
|  | AT3G26618 | Eukaryotic peptide chain release factor subunit 1-3 | 1/6 |
|  | AT3G04930 | Probable transcription factor At3g04930 | 1/6 |
|  | AT1G12920 | Eukaryotic peptide chain release factor subunit 1-2 | 1/6 |
|  | AT5G28040 | Probable transcription factor At5g28040 | 1/6 |
|  | AT5G03300 | Adenosine kinase 2 | 1/6 |
|  | AT1G06020 | Probable fructokinase-3 | 1/6 |
|  | AT3G16420 | PYK10-binding protein 1 | 1/6 |
|  | AT3G27300 | Glucose-6-phosphate 1-dehydrogenase, cytoplasmic isoform 1 | 1/6 |
|  | AT5G04430 | Protein BTR1 | 1/6 |
|  | AT5G48880 | 3-ketoacyl-CoA thiolase 5, peroxisomal | 1/6 |
|  | AT2G15430 | DNA-directed RNA polymerases II, IV and V subunit 3 | 1/6 |
|  | AT3G19980 | Phytochrome-associated serine/threonine-protein phosphatase 3 | 1/6 |
|  | AT5G03630 | Monodehydroascorbate reductase 2 | 1/6 |
|  | AT1G43670 | Fructose-1,6-bisphosphatase, cytosolic | 1/6 |
|  | AT4G29830 | WD repeat-containing protein VIP3 | 1/6 |
|  | AT1G75280 | Isoflavone reductase homolog P3 | 1/6 |
|  | AT3G52560 | Ubiquitin-conjugating enzyme E2 variant 1D | 1/6 |
|  | AT2G31390 | Probable fructokinase-1 | 1/6 |
|  | AT3G59480 | Probable fructokinase-4 | 1/6 |
|  | AT2G15400 | DNA-directed RNA polymerases IV and V subunit 3B | 1/6 |
|  | AT1G50370 | Phytochrome-associated serine/threonine-protein phosphatase 1 | 1/6 |
|  | AT2G24200 | Leucine aminopeptidase 1 | 1/6 |
|  | AT5G44316 | Putative UPF0051 protein ABCI9 | 1/6 |
|  | AT4G27520 | Early nodulin-like protein 2 | 1/6 |
|  | AT5G43330 | Malate dehydrogenase 2, cytoplasmic | 1/6 |
|  | AT2G33340 | Pre-mRNA-processing factor 19 homolog 2 | 1/6 |
|  | AT3G48500 | Protein PLASTID TRANSCRIPTIONALLY ACTIVE 10 | 1/6 |
|  | AT1G53310 | Phosphoenolpyruvate carboxylase 1 | 1/6 |
|  | AT5G03690 | Fructose-bisphosphate aldolase 4, cytosolic | 1/6 |
|  | AT3G62830 | UDP-glucuronic acid decarboxylase 2 | 1/6 |
|  | AT2G01520 | MLP-like protein 328 | 1/6 |
|  | AT2G47650 | UDP-glucuronic acid decarboxylase 4 | 1/6 |
|  | AT3G53520 | UDP-glucuronic acid decarboxylase 1 | 1/6 |
| α-TeP1 |  | TEP1 | 4/6 |
|  | AT5G49460 | ATP-citrate synthase beta chain protein 2 | 2/6 |
|  | AT4G04770 | UPF0051 protein ABCI8, chloroplastic | 2/6 |
|  | AT5G44316 | Putative UPF0051 protein ABCI9 | 2/6 |
|  | AT4G18465 | Probable pre-mRNA-splicing factor ATP-dependent RNA helicase DEAH9 | 2/6 |
|  | AT1G72730 | Eukaryotic initiation factor 4A-3 | 1/6 |
|  | AT3G02040 | Glycerophosphodiester phosphodiesterase GDPD1, chloroplastic | 1/6 |
|  | AT3G56070 | Peptidyl-prolyl cis-trans isomerase CYP19-3 | 1/6 |
|  | AT4G26250 | Galactinol synthase 6 | 1/6 |
|  | AT1G09090 | Respiratory burst oxidase homolog protein B | 1/6 |
|  | AT3G44300 | Nitrilase 2 | 1/6 |
|  | AT5G63470 | Nuclear transcription factor Y subunit C-4 | 1/6 |
|  | AT3G55620 | Eukaryotic translation initiation factor 6-2 | 1/6 |
|  | AT3G01910 | Sulfite oxidase | 1/6 |
|  | AT2G39770 | Mannose-1-phosphate guanylyltransferase 1 | 1/6 |
|  | AT4G05420 | DNA damage-binding protein 1a | 1/6 |
|  | AT4G24190 | Endoplasmin homolog | 1/6 |
|  | AT5G47890 | NADH dehydrogenase [ubiquinone] 1 alpha subcomplex subunit 2 | 1/6 |
|  | AT5G15810 | Probable tRNA (guanine(26)-N(2))-dimethyltransferase 1 | 1/6 |
|  | AT5G41220 | Glutathione S-transferase T3 | 1/6 |
|  | AT1G77760 | Nitrate reductase [NADH] 1 | 1/6 |
|  | AT2G26990 | COP9 signalosome complex subunit 2 | 1/6 |
|  | AT3G61220 | (+)-neomenthol dehydrogenase | 1/6 |
|  | AT1G54830 | Nuclear transcription factor Y subunit C-3 | 1/6 |
|  | AT3G48590 | Nuclear transcription factor Y subunit C-1 | 1/6 |
|  | AT1G08970 | Nuclear transcription factor Y subunit C-9 | 1/6 |
|  | AT5G41210 | Glutathione S-transferase T1 | 1/6 |
|  | AT2G24190 | Short-chain dehydrogenase/reductase 2b | 1/6 |
|  | AT4G04800 | Peptide methionine sulfoxide reductase B3 | 1/6 |
|  | AT4G21850 | Peptide methionine sulfoxide reductase B9 | 1/6 |
|  | AT5G20830 | Sucrose synthase 1 | 1/6 |
|  | AT1G36160 | Acetyl-CoA carboxylase 1 | 1/6 |
|  | AT3G06650 | ATP-citrate synthase beta chain protein 1 | 1/6 |
|  | AT1G72550 | Phenylalanine--tRNA ligase beta subunit, cytoplasmic | 1/6 |
|  | AT1G73250 | GDP-L-fucose synthase 1 | 1/6 |
|  | AT1G78370 | Glutathione S-transferase U20 | 1/6 |
|  | AT3G44320 | Nitrilase 3 | 1/6 |
|  | AT5G42980 | Thioredoxin H3 | 1/6 |
|  | AT1G31812 | Acyl-CoA-binding domain-containing protein 6 | 1/6 |
|  | AT1G08830 | Superoxide dismutase [Cu-Zn] 1 | 1/6 |
|  | AT5G40370 | Glutaredoxin-C2 | 1/6 |
|  | AT1G27860 | UPF0725 protein At1g27860 | 1/6 |
|  | AT4G34870 | Peptidyl-prolyl cis-trans isomerase CYP18-4 | 1/6 |
|  | AT4G29830 | WD repeat-containing protein VIP3 | 1/6 |
