# Supplementary material for: Investigation of the effects of P1 on HC-pro-mediated gene silencing suppression through genetics and omics approaches
Source: Bot Stud. 2020 Aug 3;61:22. doi: 10.1186/s40529-020-00299-x (PMC7399735; doi:10.1186/s40529-020-00299-x)
Supplement: Supplementary file 3 — Additional file 3: Table S2. Critical genes in Col-0 vs. P1/HCR network. [file 40529_2020_299_MOESM3_ESM.docx]

**Additional file 3: Table S2. Critical genes in Col-0 vs. *P1/HC^R^* network**

| Function | AGI | Gene Name | Description |
| --- | --- | --- | --- |
| Gene silencing & RNA regulation | AT1G48410 | *AGO1* | ARGONAUTE |
|  | AT1G31280 | *AGO2* |  |
|  | AT1G31290 | *AGO3* |  |
|  | AT3G44260 | *CAF1A* | CCR4-ASSOCIATED FACTOR |
|  | AT5G22250 | *CAF1B* |  |
|  | AT5G39660 | *CDF2* | CYCLING DOF FACTOR 2 |
| Calmodulin (CAM) | AT4G27280 | *CMI1* | CA2+-DEPENDENT MODULATOR OF ICR1 |
|  | AT4G34150 | *CaLB* | Calcium-dependent lipid-binding |
|  | AT5G66210 | *CPK28* | Calcium-dependent protein kinase |
|  | AT5G26920 | *CBP60G* | CAM-binding |
|  | AT2G41010 | *CBP25* |  |
|  | AT1G76650 | *CML38* | CALMODULIN-like |
|  | AT3G25600 | *CML16* | Calcium-dependent protein kinase |
|  | AT5G37770 | *CML24* | CAM-binding |
|  | AT2G30360 | *CIPK11* | CBL-interacting protein kinase |
| ETHYLENE | AT4G17500 | *ERF1* | Ethylene responsive element binding factor |
|  | AT3G15210 | *ERF4* |  |
|  | AT5G47230 | *ERF5* |  |
|  | AT4G17490 | *ERF6* |  |
|  | AT5G51190 | *ERF105* |  |
|  | AT5G61600 | *ERF104* |  |
|  | AT1G28360 | *ERF12* |  |
|  | AT4G11280 | *ACS6* | ACC synthase |
|  | AT4G17230 | *SCL13* | SCARECROW-like |
| Jasmonate | AT1G32640 | *JAI1* | JASMONATE INSENSITIVE 1 |
|  | AT1G17380 | *JAZ5* | JASMONATE-ZIM-DOMAIN |
|  | AT1G19180 | *JAZ1* |  |
|  | AT2G46510 | *JAM1* | JA-ASSOCIATED MYC2-LIKE |
|  | AT1G61340 | *FBS1* | F-BOX STRESS INDUCED 1 |
|  | AT1G80840 | *WRKY40* | WRKY TF |
| Defense related | AT1G72940 | TIR | Toll-interleukin-resistance (TIR) domain |
|  | AT1G63750 | TIR-NBS-LRR |  |
|  | AT5G41750 | TIR-NBS-LRR |  |
|  | AT5G41740 | TIR-NBS-LRR |  |
|  | AT5G58120 | TIR-NBS-LRR |  |
|  | AT1G66090 | TIR-NBS-LRR |  |
|  | AT1G72950 | TIR-NBS-LRR |  |
|  | AT4G37250 | LRRK | Leucine-rich repeat protein kinase |
|  | AT1G78780 |  | Pathogenesis-related family protein |
|  | AT3G11650 | *NHL2* | Disease resistance protein |
|  | AT5G38280 | *PR5K* | PR5-like receptor kinase |
|  | AT5G45110 | *NPR3* | NPR1-like protein |
|  | AT1G29690 | *CAD1/NSL2* | Constitutively activated cell death 1/ Necrotic spotted lesion 2 |
|  | AT5G04720 | *ADR1-L2* | ADR1-like 2 |
|  | AT3G11840 | *PUB24* | PLANT-U-BOX 24 |
|  | AT2G22880 | VQ | VQ motif |
|  | AT1G67470 | *ZRK12* | Protein kinase |
|  | AT3G16720 | *TL2* | TOXICOS EN LEVADURA 2 |
|  | AT5G18270 | *NAC87* | NAC DOMAIN CONTAINING PROTEIN |
|  | AT5G59820 | *ZAT12/RHL41* | RESPONSIVE TO HIGH LIGHT 41 |
|  | AT1G33560 | *ADR1* | ACTIVATED DISEASE RESISTANCE 1 |
|  | AT1G27730 | *STZ/ZAT10* | Zinc finger |
| ABA | AT3G51895 | *SULTR3;1* | SULFATE TRANSPORTER 3;1 |
|  | AT5G58900 | *DIV1* | DIVARICATA |
|  | AT5G04760 | *DIV2* |  |
| Auxin | AT3G49670 | *BAM2* | BARELY ANY MERISTEM 2 |
|  | AT2G33860 | *ARF3* | Auxin response transcription factor |
|  | AT5G37020 | *ARF8* |  |
| MiRNA Target | AT2G22840 | *GRF1* | Growth-regulating factor |
|  | AT1G08830 | *SOD1* | Superoxide dismutase |
|  | AT2G28190 | *SOD2* |  |
|  | AT1G12520 | *CSS1* | **COPPER CHAPERONE FOR SOD1** |
|  | AT2G33770 | *PHO2* | PHOSPHATE 2 |
|  | AT1G52150 | *ATHB-15/ICU4* | HD-ZIP domain |
|  | AT1G24260 | *SEP3* | AGAMOUS-like |
|  | AT2G34710 | *PHB* | PHABULOSA |
|  | AT5G60120 | *TOE2* | TARGET OF EARLY ACTIVATION TAGGED (EAT) 2 |
|  | AT5G50570 | *SPL13A* | SQUAMOSA PROMOTER-BINDING PROTEIN LIKE 13 |
|  | AT5G50670 | *SPL13B* |  |
